# Comparative physiological and biochemical mechanisms in diploid, triploid, and tetraploid watermelon (*Citrullus lanatus* L.) grafted by branches

**DOI:** 10.1038/s41598-023-32225-z

**Published:** 2023-03-27

**Authors:** Mohamed Omar Kaseb, Muhammad Jawad Umer, Xuqiang Lu, Nan He, Muhammad Anees, Eman El-remaly, Ahmed Fathy Yousef, Ehab A. A. Salama, Hazem M. Kalaji, Wenge Liu

**Affiliations:** 1 Zhengzhou Fruit Research Institute, Chinese Academy of Agricultural Sciences, Henan Joint International Research Laboratory of Fruits and Cucurbits Biological Science in South Asia, Zhengzhou, 450009 China; 2 Cross Pollenated Plants Department, Horticulture Research Institute, Agriculture Research Center, Giza, 12611 Egypt; 3 State Key Laboratory of Cotton Biology/Institute of Cotton Research, Chinese Academy of Agricultural Sciences (ICR, CAAS), Anyang, 455000 China; 4 Present Address: Department of Horticulture, College of Agriculture, University of Al-Azhar (Branch Assiut), Assiut, 71524 Egypt; 5 Agricultural Botany Department, Faculty of Agriculture Saba Basha, Alexandria University, Alexandria, 21531 Egypt; 6 Department of Plant Biotechnology, Centre for Plant Molecular Biology and Biotechnology, TNAU, Coimbatore, 641003 India; 7 Department of Plant Physiology, Institute of Biology, Warsaw University of Life Sciences SGGW, Warsaw, Poland; 8 Institute of Technology and Life Sciences, National Research Institute, Falenty, Al. Hrabska 3, 05-090 Raszyn, Poland

**Keywords:** Biochemistry, Genetics, Molecular biology

## Abstract

Seed production for polyploid watermelons is costly, complex, and labor-intensive. Tetraploid and triploid plants produce fewer seeds/fruit, and triploid embryos have a harder seed coat and are generally weaker than diploid seeds. In this study, we propagated tetraploid and triploid watermelons by grafting cuttings onto gourd rootstock (*C. maxima* × *C. mochata*). We used three different scions: the apical meristem (AM), one-node (1N), and two-node (2N) branches of diploid, triploid, and tetraploid watermelon plants. We then evaluated the effects of grafting on plant survival, some biochemical traits, oxidants, antioxidants, and hormone levels at different time points. We found significant differences between the polyploid watermelons when the 1N was used as a scion. Tetraploid watermelons had the highest survival rates and the highest levels of hormones, carbohydrates, and antioxidant activity compared to diploid watermelons, which may explain the high compatibility of tetraploid watermelons and the deterioration of the graft zone in diploid watermelons. Our results show that hormone production and enzyme activity with high carbohydrate content, particularly in the 2–3 days after transplantation, contribute to a high survival rate. Sugar application resulted in increased carbohydrate accumulation in the grafted combination. This study also presents an alternative and cost-effective approach to producing more tetraploid and triploid watermelon plants for breeding and seed production by using branches as sprouts.

## Introduction

Seedless watermelon varieties are preferred and accepted by consumers, and have a higher price and better quality than seeded watermelons (2n)^1^. The seedless watermelon [triploid (3n)] is produced by crossing between a tetraploid (4n) seed parent with a seeded watermelon [diploid (2n)]^2^. Tetraploid induction can be achieved using various methods, such as applying aqueous chemical solutions (such as colchicine, oryzaline, and dinitroaniline) to the growing tips of diploid seedlings, or by soaking diploid seeds before germination^3^. However, these chemicals are toxic, which reduces the success rate^4^. Herbal grafting is used commercially because it offers a wide range of essential benefits such as enhancing the resistance to biotic and abiotic stresses along with yield improvement^5–7^. In contrast, soil quality degrades over time due to successive land use, while stress and disease occur more frequently, thereby improving plant growth, and increasing yields^8^. Successful grafting is a complex biochemical and structural process that involves initial wound reaction, callus formation, cambium formation, and the development of a functional vasculature between scion and rootstock^9^. All of these steps can shape the future of a grafted plant^10^. However, there are no general rules about how these treatments affect incompatibility responses in tolerance reactions^11,12^. To date, the causes and underlying mechanisms of grafting incompatibility remain elusive, and there are no reports on genes associated with grafting incompatibility^13,14^. More research is needed to fully understand graft compatibility and incompatibility mechanisms^15,16^.

Graft incompatibility in fruit trees occurs due to the accumulation of various factors^17^. Pina et al.^18^ found that reduced auxin and lignification, which are associated with high phenols in incompatible grafts, could indicate weak graft junctions, reactive oxygen species (ROS)^19–22^, or hormone imbalance^11,23,24^. IAA and cytokinin play vital roles in regulating rootstock/scion interactions^25^, and are necessary for vascular bundle regeneration at the graft union^26,27^, the differentiation of vascular tissues and wound healing^21,22,28–31^. There are three main classes of auxin-responsive genes: the GH3, Aux/IAA, and SAUR gene families^32–35^. Liu et al.^36^ found that *ARF1, ARF8, GH3,* and *IAA4* expression levels were shown to be negatively correlated with growth vigor and IAA content. Also, the differential expression of *KO1* and *GA2OX1* in grafted trees had an impact on the metabolism of GA.

During the healing process of grafting, the grafted plants are exposed to various stresses, such as injuries or wounds, total darkness, and high humidity^15,37^. The second and third days after grafting are crucial for the healing process^38^. In shoots of apple grafted on a dwarf rootstock, Li and colleaguesdiscovered that the expression of the gene *PIN1*, which is related to polar auxin transport, was markedly reduced^39^. The alteration in gene expression led to a decrease in the top-down polar transport of indole-3-acetic acid (IAA), which caused inadequate delivery of IAA to the apple roots.From Ren and colleages, it was discovered that *CmRNF5* and *CmNPH3L* are variably expressed across compatible and incompatible graft unions throughout graft development of cucumber/pumpkin graft’s growth^19^. These two genes may be associated with transplant compatibility/incompatibility responses based on the difference in expression of the two genes seen in the scion and various compatibility records. The results showed that both genes are affected by stress. The accumulation of reactive oxygen species (ROS) leads to cell death, and oxidative damage is due to the imbalance between the production of antioxidants (AOX) and ROS^40,41^. Increasing the activity of defense enzymes such as peroxidase (POD) and catalase (CAT) can scavenge ROS in plants and improve the resistance of plants to stress^42,43^. Guaiacol peroxidase (POX) and CAT convert H_2_O_2_ to H_2_O^44^. So, the differences between AOX and ROS during the healing process could be used as a rapid mechanism to verify incompatibility^45^. Lignin is abundant in woody plants and primarily contributes strength to the cell wall such that reduced lignin would counteract strong graft union^16^. Most antioxidant enzyme genes were upregulated in leaves from trees grafted onto red tangerine, resulting in higher POD activity^36^. In addition, studies have found differences in gene expression studied rootstock/sprout combinations of sweet cherry^46^ and grape^47^.

Furthermore, carbohydrates play an important role in the cellular activities of plants by providing energy^48,49^. The development of graft union in the grafting compound primarily depends on the levels of carbohydrates present in the rootstock and scion during the grafting process. Higher levels of carbohydrates, along with auxins and cytokines, are crucial for the formation of effective callus. Interactions between rootstock and scion cultivars of watermelon for carbohydrate utilization can affect the starch degradation that occurs throughout the grafting process; the survival rate was positively correlated with increased starch content^49^.

Previous research on perennial crops has shown that increasing carbohydrate content can increase the success rate of grafting. For instance, in grapes, there is a positive correlation between callus formation and carbohydrate content in grafted grapes, leading to higher survival rates of the transplanted material^50^. Likewise, studies on grape, macadamia, lychee, avocado, and watermelon seedlings indicated that increasing starch content in the shoot has a positive correlation with successful grafting^49–53^.

The current research aimed to study the effect of genome duplication on graft compatibility by comparing the factors and parameters that lead to graft compatibility or incompatibility. This study provides molecular and physiological level information for the compatibility mechanism in polyploid watermelons and thus explains the compatibility mechanism in polyploid watermelons using branches as scions for plant propagation. Three different branches were taken from mother plants and grafted onto squash rootstock to study compatibility and the effect of sucrose application on graft compatibility. Grafting branches will help breeders and seed producers save time and money by using an asexual method of increasing plant numbers.

## Results

### Survival rates of diploid, triploid, and tetraploid watermelons

**Figure 1:**
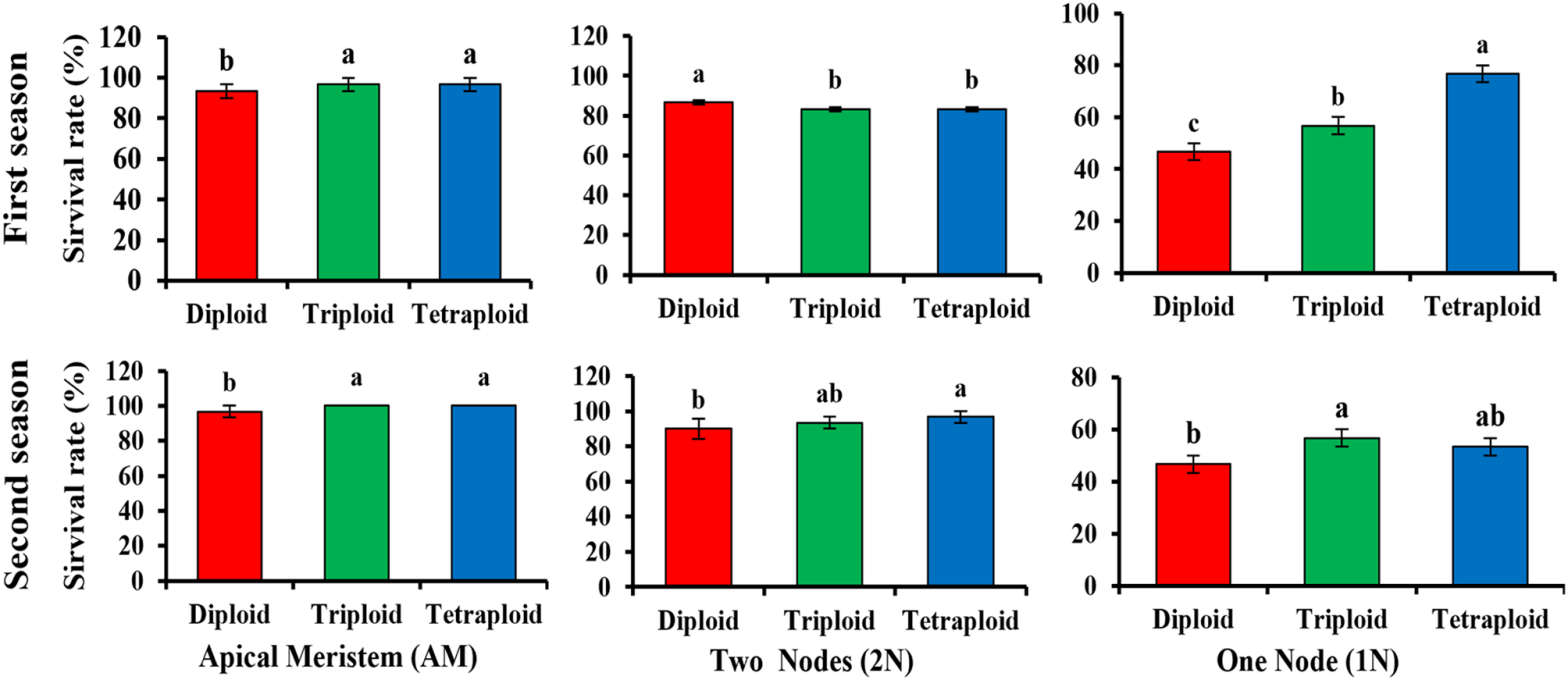
The survival rate of watermelon with different ploidy level grafted with (AM, 1N, and 2N) branches. (**A**): Apical Meristem (AM), (**B**): Branch with 1 Node (1N), (**C**): Branch with 2 Nodes (2N).

**Figure 2:**
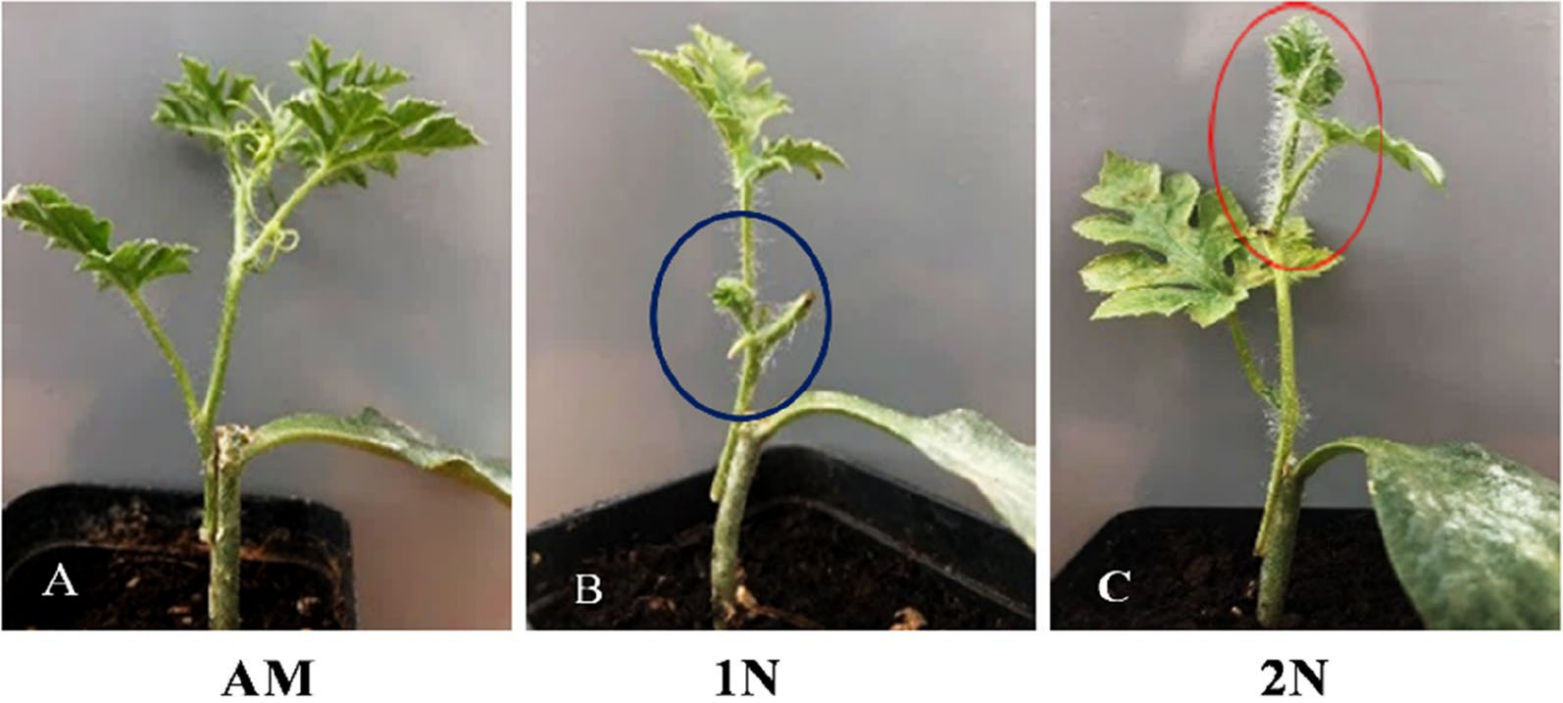
Growth patterns of grafted plants with different branches 15 days after grafting. (**A**): Growing apical meristem (AM), (**B**): Growing axillary bud in branch with 1 Node (1N), (**C**): Growing second axillary bud in branch with 2 Nodes (2N). Blue circle shows branch with one node whereas, red color indicates branch with two nodes.

Plant survival rates were recorded for all grafting combinations among diploids, triploids and tetraploids grafted from different branches of mother plants on interspecific rootstock (*C. maxima* × *C. mochata*) during two consecutive seasons (First season: August 2021 and Second season: March 2022). Statistical analysis showed significant differences between diploid, triploid and tetraploid scions when 1N was used as a scion. The combined analysis for two seasons gave 45.45, 55.45, and 78.03% in diploid, triploid and tetraploid watermelon, respectively. In the first season, grafting by AM gave the highest survival rate of 93.3, 96.67, and 96.67% in the first season and 96.67, 100, and 100% in the second season in diploid, triploid, and tetraploid watermelons, respectively. However, no significant differences were observed between polyploid scions. Also branches with two nodes (2N) showed no significant differences between polyploids in terms of survival rate of 86.67, 83.33, and 83.33% in the first season and 90, 93.33, and 96.67% in the second season in diploid, triploid, and tetraploid watermelons, respectively (Figs. 1 and 2) and the survival rate of watermelons with different degrees of ploidy after 30 DAG (Figs. S2 and S3).

Our results also showed the suitability of vegetative propagation using AM, 2N, and 1N branches as scions in tetraploid watermelon.

### Measurement of IAA and ZR in the graft combination among diploid, triploid, and tetraploid watermelon on different days after grafting

**Figure 3:**
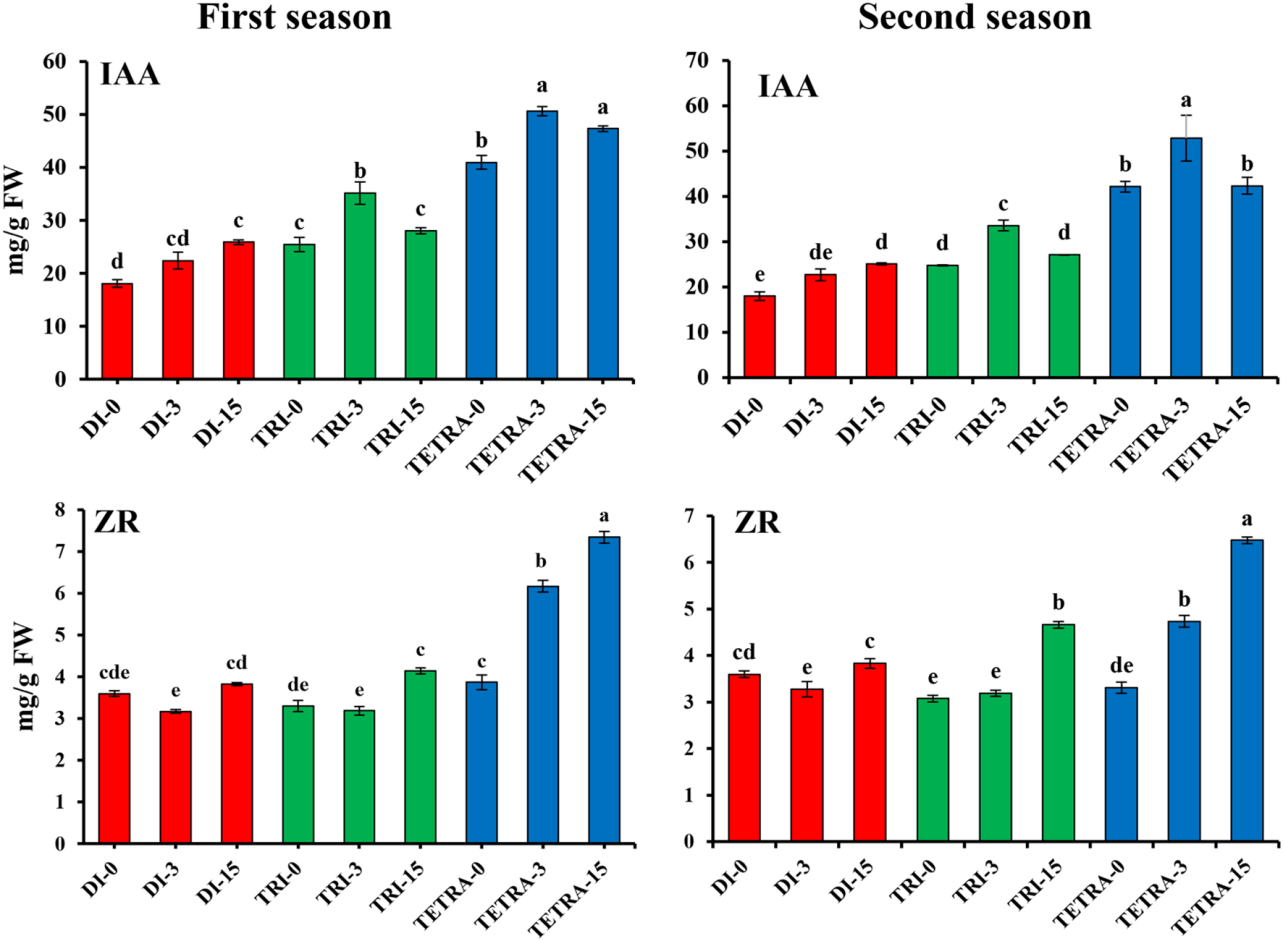
IAA and ZR contents of watermelons with different ploidy levels grafted on pumpkin rootstock at 15 DAG during August 2021 and March 2022 cropping seasons. The values are means of fifteen independent plants (n = 15). Different letters indicate significant differences at *P* < *0.01* using Duncan'ıs multiple range test. *IAA* indole acetic acid, *ZR* zeatin riboside, *Di* diploid, *Tri* triploid, *Tetra* tetraploid.

Ploidy levels (diploid, triploid and tetraploid) exerted a significant impact on the IAA content in the graft combination at all stages 0, 3 and 15 from DAG (Fig. 3). At the beginning of grafting, the IAA content of triploids and tetraploids was 2.35 and 1.4 times higher than the content of diploids in the first and second seasons, respectively. Furthermore, the increase in IAA content in diploid, triploid and tetraploid scion 3 days after grafting was 23.88, 37.93 and 23.68% in the first season and 26.35, 35.82 and 25.34% in the second season in diploid, triploid, and tetra watermelons, respectively. IAA content was higher in tetraploids 0, 3 and 15 after grafting and followed by diploids and triploids.

In contrast, ZR at 0 DAG showed no significant differences between diploid and tetraploid, while at 3 and 15 DAG there were quite significant differences in both seasons. (Fig. 3). Results 3 and 15 DAG showed that ZR content increases significantly in tetraploids than in diploids and triploids. In 3 DAG, ZR content in tetraploid watermelon plants increased by 59.43% and 42.9% at 15 DAG by 18.69 and 37%, respectively, during the first and second season, respectively.

### Measurement of antioxidants (POD, SOD, and CAT) and H_2_O_2_ in the graft combination among diploid, triploid, and tetraploid watermelon on different days after grafting

**Figure 4:**
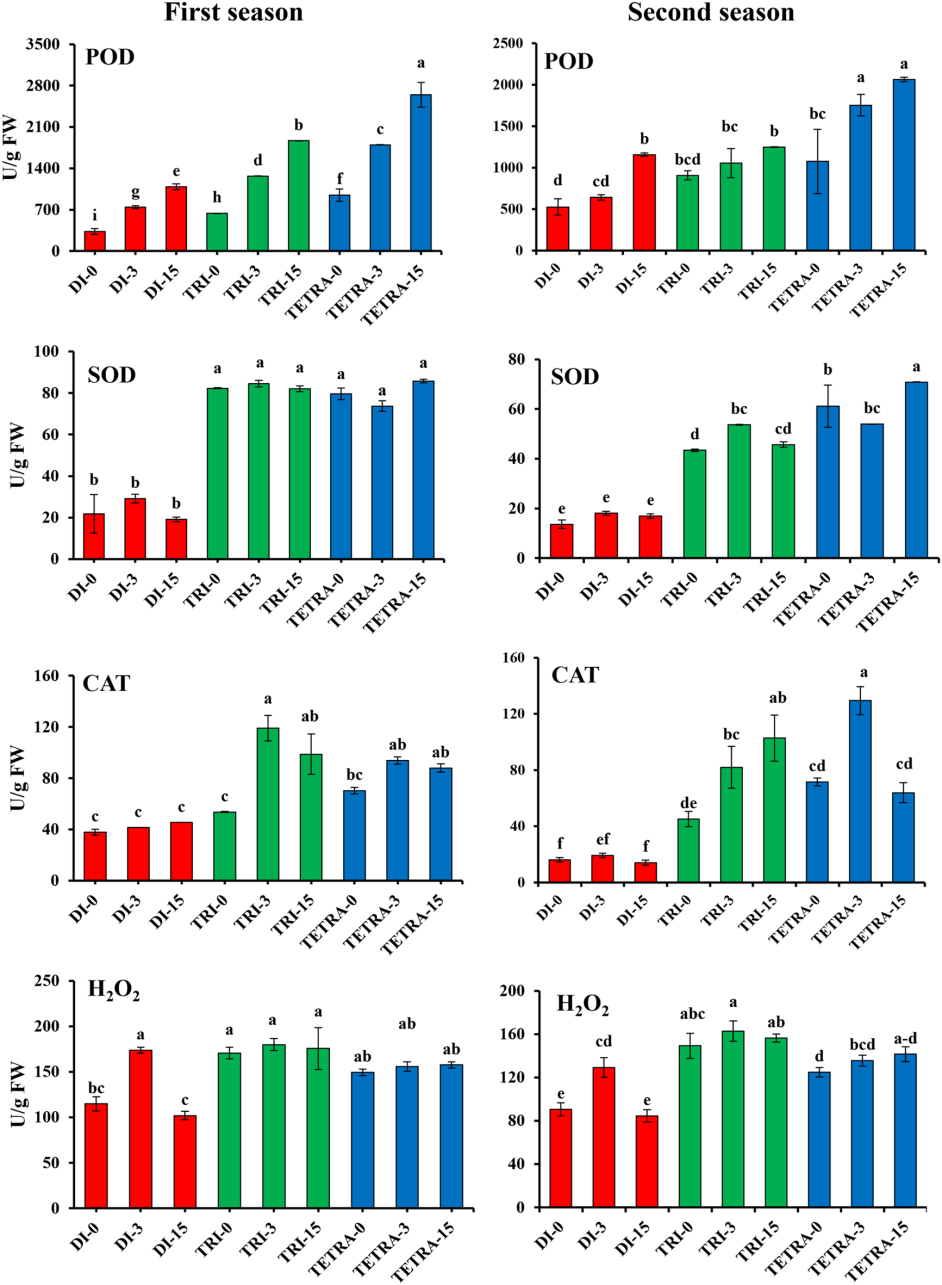
POD, SOD, and CAT activities in watermelons with different ploidy levels grafted on pumpkin rootstock at 0, 3, and 15DAG during August 2021 and March 2022 cropping seasons. The values are means of 15 independent plants (n = 15). Different letters indicate significant differences at *P* < *0.01* using Duncan'ıs multiple range test. *POD* peroxidase, *SOD* superoxide dismutase, *CAT* catalase, *Di* diploid, *Tri* triploid, *Tetra* tetraploid.

In general, POD enzyme activity showed an increased tendency following grafting (Fig. 4). The highest POD activities were found in tetraploid plants at the beginning of grafting with 2.87 and 2.05 fold compared to diploid and 1.48 and 1.19 fold compared to triploid in the first and second season, respectively. At 3 DAG, it was observed that POD activities in tetraploid was higher than in diploid and triploid plants, while the POD activities in diploid, triploid, and tetraploid watermelon was 124.84, 98.88, and 89.83% in the first season and 22.34, 16.25, and 63.21% during the second season, respectively. While at 15 DAG, the growth rates were (46.86, 47.04, and 47.09%) in the first season and 80.53, 18.64, and 17.74% in the second season in diploid, triploid and tetraploid watermelon, respectively.

At the beginning of grafting (0 DAG), higher SOD activity was observed in tetraploid and triploid watermelons compared to diploid watermelons (Fig. 4). The activities of SOD were 3.76 and 3.19-fold higher in triploid than diploid watermelons, respectively, at the beginning of grafting in both seasons. At 3 DAG, the SOD activity in triploid watermelons was 2.9 and 2.97-fold higher than in diploid watermelons in the first and second seasons, respectively. At 15 DAG, the SOD activity was 4.32-fold higher in triploid watermelon plants than in diploid watermelon plants. Generally, there were significant differences between tetraploid and diploid plants at 0, 3, and 15 DAG, and the SOD contents in tetraploid and triploid plants were higher than in diploid plants. Additionally, a significantly higher CAT activity was observed in tetraploids compared to diploids and triploids (Fig. 4). At the beginning of grafting (0 DAG), the CAT activity in tetraploids was 1.85 and 2.35-fold higher than in diploids and 1.31 and 1.7-fold higher than in triploids in the first and second season, respectively. At 3 DAG, the CAT activity in graft combinations began to increase in tetraploid and triploid plants compared to diploid plants. The CAT activity in diploid, triploid, and tetraploid watermelon plants was 19.55%, 81.42%, and 81.15%, respectively. At the beginning of grafting, the H_2_O_2_ content was higher in triploid plants than in diploid and tetraploid plants (Fig. 4).We observed that the H_2_O_2_ content in triploid watermelon plants was 1.49 and 1.65-fold higher than diploid and 1.14 and 1.2-fold higher than tetraploid plants in the first and second seasons, respectively. However, the increment rates of H_2_O_2_ activity in the graft combination began to increase in tetraploid plants at 3 DAG, but to a lesser extent than in diploid and triploid plants. The growth rates were 51.51%, 5.43%, and 4.08% in the first season and 42.53%, 8.62%, and 8.79% in the second season for diploid, triploid, and tetraploid plants, respectively. This may be attributed to the higher activity of AOX in tetraploid plants compared to diploid and triploid plants.

### Measurement of lignin, phenols, and starch contents in the graft combination among diploid, triploid, and tetraploid watermelon on different days after grafting

**Figure 5:**
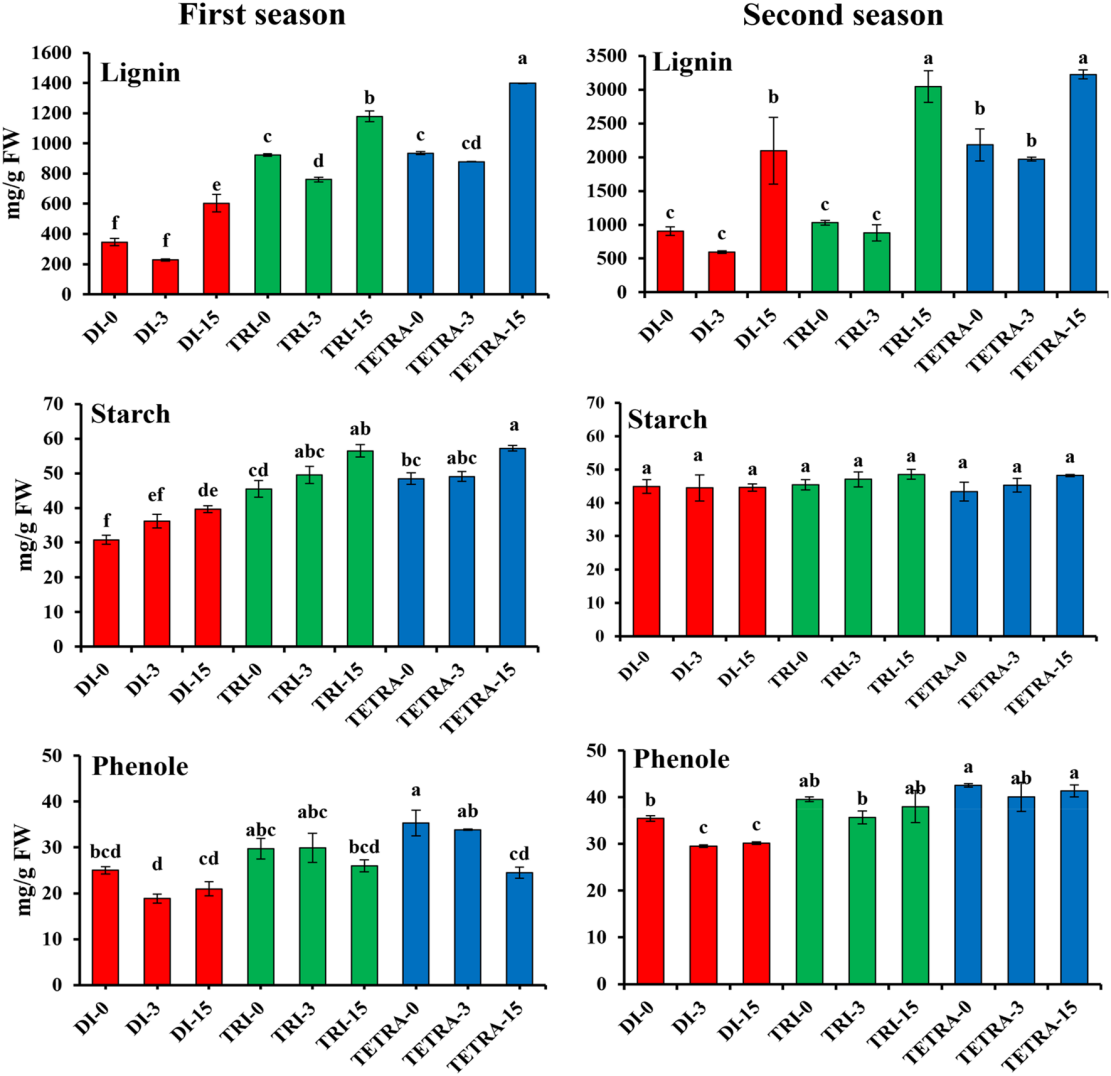
Lignin, phenols, and starch contents of watermelons with different ploidy levels grafted on pumpkin rootstock 0, 3, and 15 days after grafting during August 2021 and March 2022 cropping seasons. The values are means of fifteen independent plants (n = 15). Different letters indicate significant differences at *P* < *0.01* using Duncan'ıs multiple range test. *Di* diploid, *Tri* triploid, *Tetra* tetraploid.

The lignin content was significantly different between diploid and tetraploid watermelon plants in both seasons (Fig. 5). The lignin content was significantly different between diploid and tetraploid watermelon plants in both seasons (Fig. 5). Lignin contents were observed to be higher in triploid and tetraploid plants compared to diploid plants at the beginning of grafting. The lignin content in tetraploid plants was 2.7- and 2.41-fold higher than in diploid plants in the first and second seasons, respectively. Additionally, it was 2.67- and 2.41-fold higher in triploid plants in the first and second seasons, respectively, compared to diploid plants.At the 3 DAG stage, callus induction in the graft combination (where scion and rootstock interlocked) caused a decrease in lignin contents. However, this decrement was less pronounced in tetraploid plants compared to diploid plants.The rates of increase in lignin content in the graft combination at 3 DAG were 51.68, 21.5 and 6.33% in the first season and 51.68, 16.98 and 12.32% in the second season in diploid, triploid and tetraploid watermelon plants, respectively. The lignin content of the grafted diploid, triploid and tetraploid watermelon plants increased 164.32, 55.08 and 59.18% in the first season and 251.37, 264 and 188.11% in the second season at 15 DAG, respectively.

We observed significant differences in phenol content in diploid and tetraploid watermelons in both seasons at 3 DAG (Fig. 5). The contents of phenols in tetraploid watermelon plants were 1.4, 1.7, and 1.1-fold higher than diploid ones at 0, 3, and 15 DAG in the first season and 1.1, 1.3, and 1.2-fold at 0, 3 and 15 DAG in the second season, respectively.

Similarly, we observed significant variations in starch content in ploidy-level grafted watermelons in the first season, but no significant differences were observed in the second season (Fig. 5). The results showed that starch levels were higher in triploid and tetraploid plants than in diploid ones. At that the beginning of grafting, the starch content was 1.57 and 1.48-folds higher in triploid and tetraploid as compared to diploid plants in the first season, while in the second season there were no significant differences between the degrees of polyploidy levels. The starch contents start to increase in all watermelons with different ploidy levels 15 DAG. However, growth rates were higher in triploid (17.09%) and tetraploid (16.58%) plants than in diploid (9.68%) plants.

### Correlation of hormones, antioxidants, starch, and biochemical contents in the graft combination among diploid, triploid, and tetraploid watermelon plants on different days after grafting

**Figure 6:**
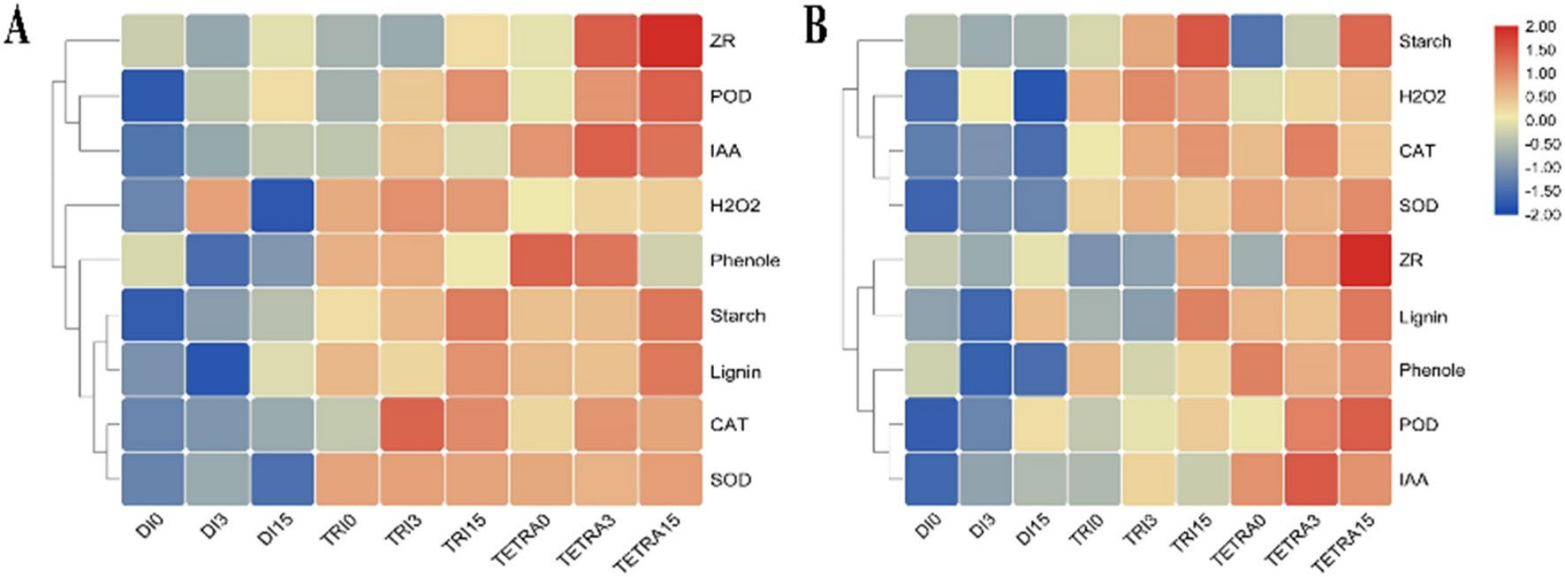
Heat map of hormones, antioxidants, starch, and biochemical contents in the grafting union at different stages in polyploid watermelon. The values of log2 fold change are shown in the heat map. (**A**) first season, (**B**) second season, *Di* diploid, *Tri* triploid, *Tetra* tetraploid at different stages (0, 3, and 15 Days after Grafting). TBtools software (version ≥ 1.6) was used to generate this figure and it is freely available to non-commercial users at https://github.com/CJ-Chen/TBtools/releases.

The heat map (A and B) in the first and second seasonal visualization of all hormones and biochemicals (Fig. 6) showed a widespread change in the concentrations in the grafting union at different stages of the polyploid watermelon. Notably, the majority of compounds showed stage-related changes in abundance, either increasing or decreasing, across different polyploid levels.

### Phylogenetic analysis of IAA, ZR, antioxidants, lignin, phenols, starch metabolism, and transport genes in the graft combination among diploid, triploid, and tetraploid watermelon

BlastP searches help us to explore the watermelon genome database using protein sequences of genes associated with H_2_O_2_, starch, POD, SOD, lignin, phenols, ZR, IAA, and CAT selected from *Arabidopsis thaliana*, *Citrullus colocynthis*, *Cucumis melo*, *Cucumis sativus*, *Cucurbita maxima*, *Cucurbita moschata*, *Cucurbita pepo*, *Luffa aegyptiaca* and *Momordica charantia*, as a query to identify homologous genes in watermelons. Homologous genes in watermelons are summarized in (Fig. S4 and Table S1).

### RT-qPCR analysis of genes regulating hormones, biochemical substances, and antioxidants in diploid, triploid, and tetraploid watermelon on different days after grafting

**Figure 7:**
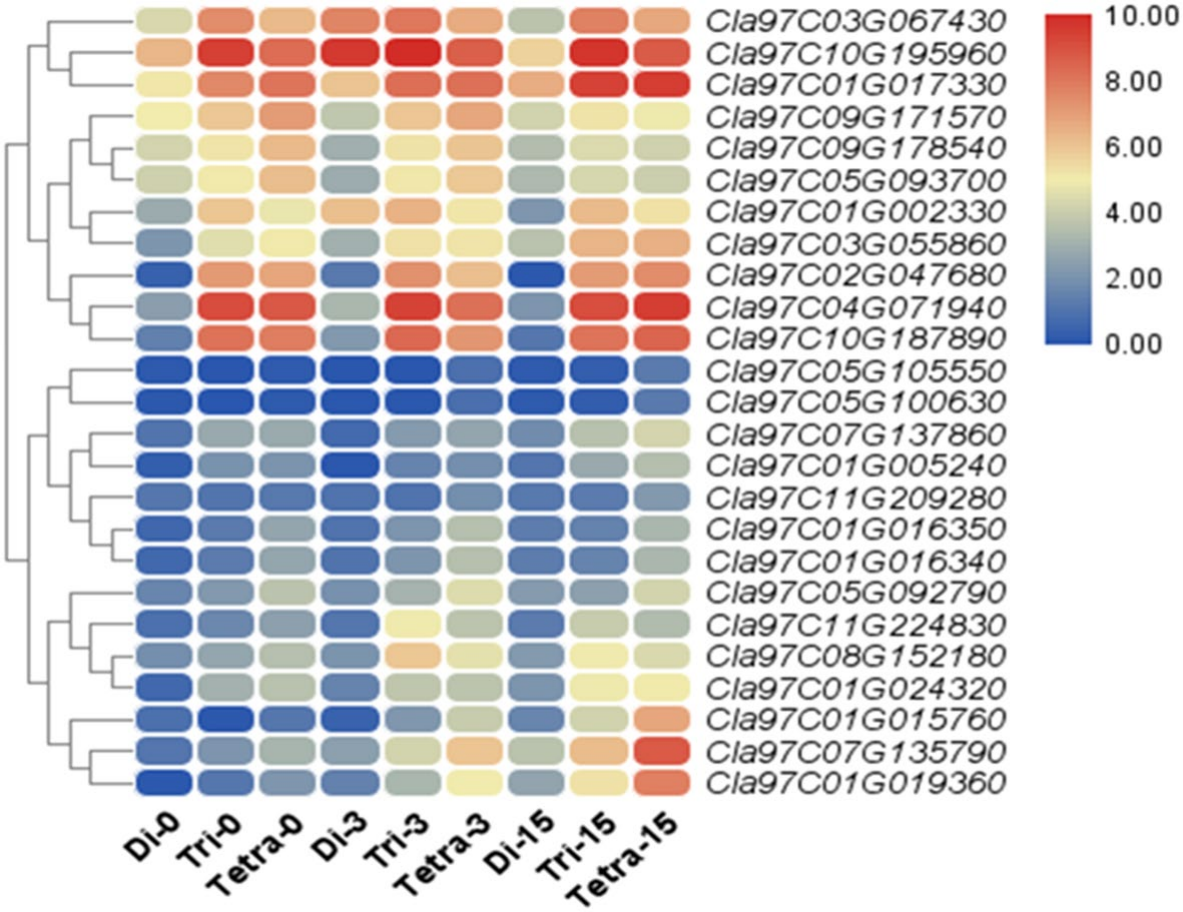
Heatmap of selected genes in grafted diploid, triploid, and tetraploid watermelons 0, 3, and 15 DAG. *Di* diploid, *Tri* triploid, *Tetra* tetraploid. TBtools software (version ≥ 1.6) was used to generate this figure and it is freely available to non-commercial users at https://github.com/CJ-Chen/TBtools/releases.

Expressions of genes regulating CAT, POD, SOD, H_2_O_2_, IAA, Cytokinin (ZR), phenols, lignin, and starch were checked and drawn phylogenetic trees at three stages 0, 3, 15 DAG (Fig. 7). The PCR product ranges were set between 80 and 200 bp, and the Roche LightCycler 480 II was used for amplification purposes^54,55^. Transcript levels of genes were associated with compatibility/incompatibility as they regulate important biochemical substances and antioxidants. *WM-IAA-1 (Cla97C05G092790)*, and *WM-ZR-1 (Cla97C05G105550)*, had higher expressions controlling IAA and ZR signaling in tetraploid watermelon followed by triploid and diploid watermelon during the grafting process in both seasons. *WM-POD-1 (Cla97C07G135790)*, *WM-CAT-1 (Cla97C08G152180), and WM-SOD-1 (Cla97C04G071940)* controlling POD, SOD, and CAT synthesis had a higher expression in tetraploid as compared to triploid and diploid watermelon. *WM-H*_*2*_*O*_*2*_*-1 (Cla97C10G195960)* controlling H_2_O_2_ production, had a lower expression level in tetraploid watermelons than in triploid and diploid watermelon plants during the grafting process. Genes regulating starch and lignin contents in watermelon, including *WM-Starch-1 (Cla97C01G017330)*, *WM-Lignin-1 (Cla97C07G137860),* controlling starch and lignin production, had higher expressions in tetraploid watermelon as compared to triploid and diploid watermelon confirming their active roles in starch and lignin accumulation at all the time points of sampling. Among the genes controlling phenol contents, *WM-Phenol-1 (Cla97C09G171570)* ion had higher expression in triploid and diploid watermelon as compared to tetraploid watermelon during the grafting process.

### Survival rates of diploid, triploid, and tetraploid watermelon after sucrose drench with different concentrations on rootstocks seedlings

**Figure 8:**
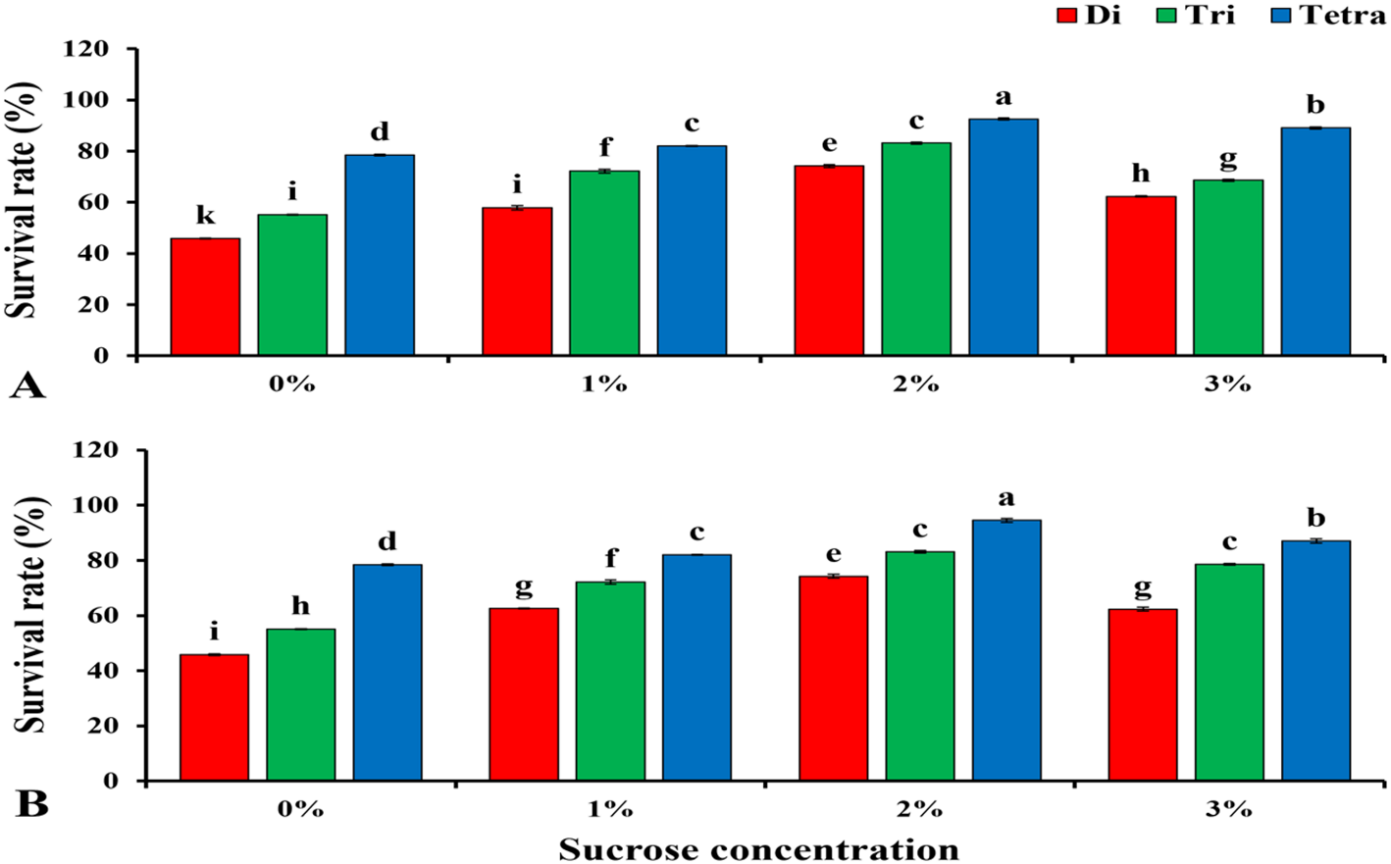
Effect of sucrose application (0, 1, 2, 3% concentrations) on the survival rate of watermelon with different ploidy levels grafted by 1 N in first season (**A**) and second season (**B**) at 15 DAG. The values are means of hundred independent plants (n = 100). Different letters indicate significant differences at *P* < *0.05* using Duncan'ıs multiple range test. *Di* diploid, *Tri* triploid, *Tetra* tetraploid.

Significant variations between polyploids were observed after sucrose application (Fig. 8). The combined analysis for two seasons with 2% sucrose application revealed that the highest survival rates in diploid, triploid and tetraploid watermelons were 74.23, 83.13 and 92.57% in the first season and 74.24, 83.13 and 94, 57% in the second season, respectively. When 3% sucrose was applied, the survival rates in diploid, triploid, and tetraploid watermelons were 62.33%, 86.6%, and 89.07% in the first season, and 62.33%, 78.6%, and 87.07% in the second season, respectively.

These results indicate that the drench applications of 2% sucrose on rootstocks seedlings before grafting could increase the survival rate when grafted by 1 N.

### Measure starch contents in the graft combinations among diploid, triploid, and tetraploid watermelon after sucrose application with different concentrations at different grafting stages

**Figure 9:**
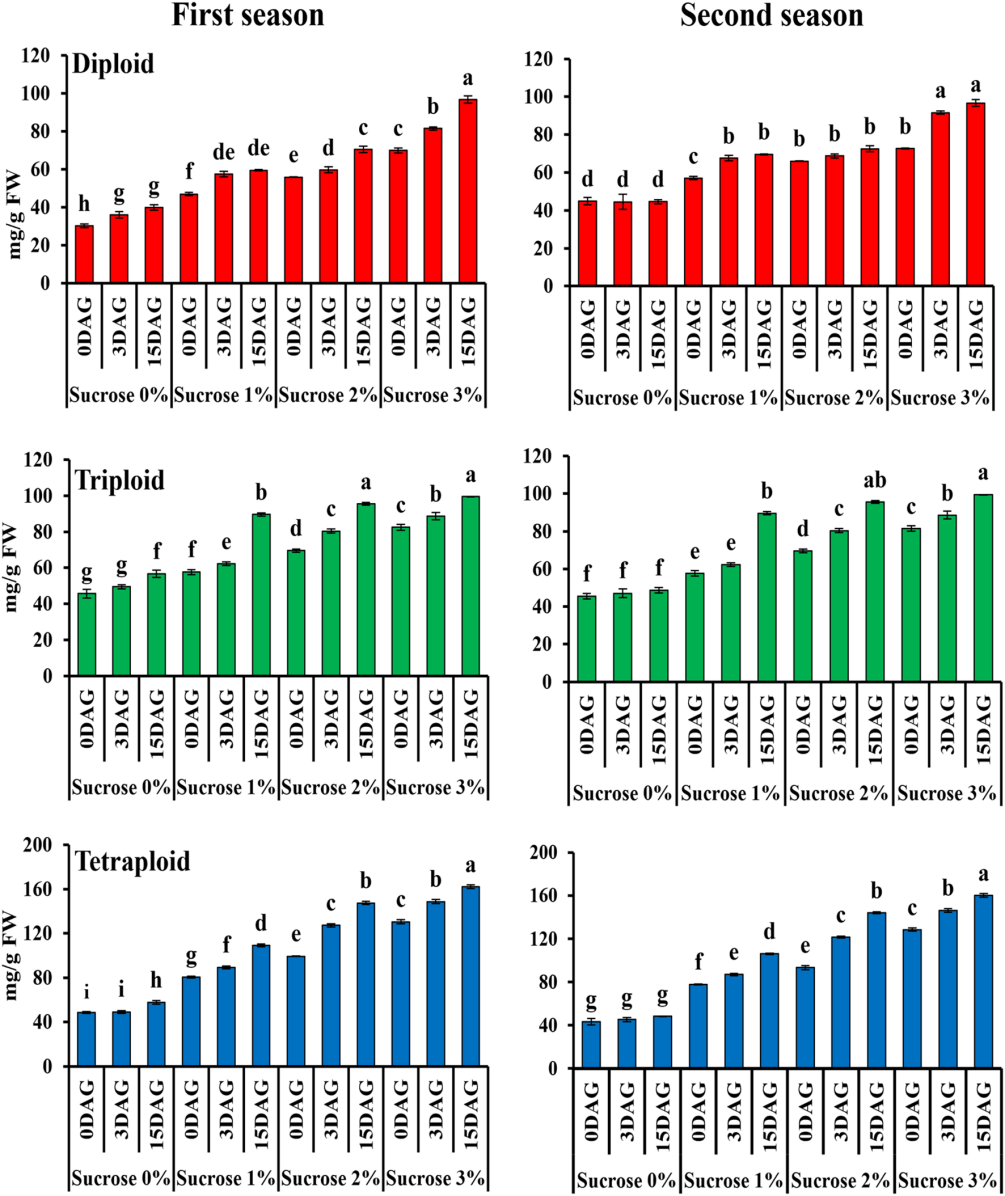
Effect of sucrose application on starch accumulation in the grafted watermelons with different ploidy level 0, 3, and 15 DAG during August 2021 and March 2022 cropping seasons. The values are means of fifteen independent plants (n = 15). Different letters indicate significant differences at *P* < *0.01* using Duncan'ıs multiple range test.

An increase in starch accumulation was observed with increasing concentration of sucrose application. Starch accumulation in grafted watermelons with different ploidy level 3 and 15 DAG was highest in plants given 3% sucrose solution than in plants given 2% sucrose solution (Fig. 9).

Compared to the control, the application of 3% sucrose led to the highest accumulation of starch. At 0, 3, and 15 days of grafting, the starch contents in the graft combinations were 131.00, 125.45, and 142.27 mg/g FW in diploids, 80.58, 85.19, and 76 mg/g FW in triploids and 168.83, 202.75, and 181.42 mg/g FW in tetraploids in the first season, respectively. While, at 0, 3 and 15 days of grafting in second season, starch contents in the graft combinations were 61.69, 105.86, and 116.9 mg/g FW in diploids, 79.23, 88.26, and 105.09 mg/g FW in triploids, and 196.35, 223.27 and 232.24 mg/g FW in tetraploids, respectively. On the other hand, the highest survival rates among all polyploids were observed with 2% sucrose application. Interestingly, the accumulation of starch in the graft combination at stage 3 DAG (critical period) was significantly higher in tetraploids than in triploids and diploids, i.e. 2.13 and 1.77-fold higher than in diploids in the first and second seasons, respectively, and 1.51-fold greater than triploid in the first and second seasons, respectively, results indicate that sucrose led to an increase in starch accumulation in grafted plants, thereby increasing survival rates in polyploid watermelons.

## Discussion

Vegetative propagation is helpful and has been applied in many other crops i.e. grapes^56^, mango^57,58^, and peach^59^, etc. to achieve good survival and avoid seed production problems. Here we grafted plants using branch cuttings as scion for successful vegetative propagation of watermelons with different ploidy levels. Triploid watermelon seeds are produced by cross-pollination between diploid and tetraploid seeds, but tetraploid seed production is arduous^60^. So, it is important to find a more economical way to increase the number and quality of triploid seeds^61^. In this study, we tested three various shoot parts of mother plants as scion, [cutting with apical meristem (AM), and branch cuttings with 1 and 2 nodes (1N and 2N)] to increase the number of mother plants to be clonally propagated by grafting technique We determined the content of hormones and antioxidants in the graft combination at 0, 3 and 15 DAG, the most critical time in the healing process is 2–3 DAG. During the healing stage, the stress is maximal (complete darkness, high humidity and temperature)^15,37^, along with an increase in hormone and antioxidant levels leading to graft compatibility^38^. Our results showed a higher survival rate (Fig. 1) with all three types of branches used for grafting into tetraploid plants, while in triploid plants grafting with AM and 2N gave higher survival rate, while grafting with 1N gave lower survival rate, but in diploid only grafting with AM gave higher survival rate. This indicates that tetraploid with a branch with 1 and 2 nodes and apical meristem, triploid with AM and 2N grafted and diploid with AM grafted are the best possible routes of vegetative propagation in watermelons.

### Accumulation of IAA and ZR in the graft combination during the grafting process

Plant hormones play important roles in plant growth, development, and response to biotic and abiotic cues and vascularization in the graft junction^15,21,22,28–30^. Auxin, and cytokinin, play an important role in regulating stock/scion interactions^25,38^. Cell divisions occurred within 2–3 DAG in the graft junction and have the highest hormone levels during this period^38,62–64^. During the grafting process, the highest IAA contents were observed within 3 DAG in the scion, as reported by Bing Song Zheng et al.^65^. IAA and ZR are required for vascular bundle regeneration in the graft union^26,27^. The main cause of incompatibility is the occurrence of hormonal imbalance^23^. A low indole-3-acetic acid (IAA) content in incompatible combinations may then affect the differentiation of xylem and phloem, as well as lignification^17,66,67^. We compared the IAA and ZR contents in the graft union between diploid, triploid, and tetraploid watermelon plants (Fig. 3). IAA contents in tetraploid graft combinations were significantly high compared to diploid and triploid combinations with *p* < *0.05*. Our results showed high compatibility in tetraploid plants, which have higher contents and growth rates of hormones than diploid plants especially at 3 DAG (critical period) and 15 DAG, these results were agreement with Cookson et al.^68^, Schaller et al.^69^, and Melnyk et al.^38^.

### Antioxidant activities in the graft combination during the grafting process

The incompatibility results from the stress induced during the healing response, as previously reported by Gainza et al.^15^. In this study (Fig. 4), the activities of POD, SOD and CAT were higher in tetraploid and triploid plants during the healing process. Also, the content of H_2_O_2_ (Fig. 4) did not increase during the healing process in triploid plants due to the high activities of antioxidants, leading to scavenging of oxygen radicals. These results were in accordance with Ganie et al.^70^ and Ruiz et al.^71^, who found that genome duplication gave high resistance to salt stress in poliploid plants due to high hormone content and antioxidant activities. Also, polyploidy leads to chromosome duplication leading to gene duplication leading to higher expression leading to an increase in protein contents^72^. Polyploids are more tolerant to abiotic stress than diploids as reported in watermelon by Zhu et al.^73^, in rice by Tu et al.^72^, in citrus by Ruiz et al.^71^, in black locust by Meng et al.^74^, in honeysuckle by Yan et al.^75^, in kinnow mandarin by Balal et al.^76^, in cotton by Said et al.^77^, and in rangpur lime by Allario et al.^78^.

Antioxidant enzymes are mainly studied in the context of abiotic and biotic stress responses, but are not often investigated in relation to engraftment stress. In the current study, we suggest that grafting represents a form of stress, including cut or wound stress, complete darkness, and high humidity stress, particularly during the first three days after grafting (healing response)^15,37^. The most critical period in the grafting healing process was at 2 and 3 days after grafting^38^. The results in both seasons showed that SOD, POD, and CAT activities were significantly different in the grafting healing process between watermelons with different ploidy levels (Fig. 4). It has already been reported by Meng et al.^43^ and Fernandez-Garcia et al.^79^ that high activities of POD and CAT during the healing process have a heightened ability to scavenge ROS and H_2_O_2_ into plants during the healing process. H_2_O_2_ is produced when the plant is injured or stressed, causing cell death^80^.

Higher enzymatic activities and accumulations of phenolic compounds are characteristically linked to plant stress resistance^81^. Phenols contribute to the elimination of ROS^82^. In our results, the content of phenols was higher at all stages than in diploid and triploid plants at all stages, thus leading to incompatibility^20,21^, and a significant decrease in phenol content was observed at later stages of grafting 15 DAG (Fig. 4). The decrement rates between 3 and 15 DAG were 38.24% in tetraploid plants, while in diploid plants the content of phenols increased at 15 DAG. In triploid plants, the decrement rates were 15% at 15 DAG. In addition, our results are similar to Xu et al.^81^, who found that the phenolic content and lignin content were higher in the compatible combination.

### Starch accumulation in the grafting combination during the grafting process

The carbohydrates provide energy in the plant and the cotyledons are a source of carbohydrates in the seedlings^49,83^. The survival rate correlated positively with an increase in starch content^53^. Our attempt to increase the proportion of carbohydrates before grafting by adding a solution of sucrose at different concentrations (0, 1, 2 and 3%) to the rootstocks led to an increase in the accumulation of starch in the plant before grafting, and this led to an increase in the survival rate, these results were identical with Dabirian and Miles^49^. We applied sucrose to the rootstock seedlings and a higher survival rate was observed in the case a 2% sucrose application followed by a 3% sucrose application in polyploid watermelon. The accumulation of starch in the graft combination was positively increased with increasing concentration of sucrose in the solution, as the treatment had the highest rate of accumulation of starch at a concentration of 3%, followed by 2% compared to the control. These results were identical with Dabirian and Miles^49^, who conducted a similar experiment to increase the survival rate in watermelons grafted by the splice grafting method. The accumulation of starch in the graft combination at stage 3 DAG (the critical period requiring the highest energy rate for callus initiation and differentiation) was significantly higher in tetraploids than in triploids and diploids. It was 2.13 times higher than diploid and 1.58 times higher than triploid. These results could explain the increase in survival rate by increasing the concentration of sucrose in the solution and could explain the increase in survival rate in tetraploids. It was higher than triploid and diploid due to its ability to store the highest rate of starch over triploid and diploid. These results are consistent with Bartolini et al.^50^, who suggested that higher carbohydrate levels in grafted tissues could lead to successful grafting. According to recent results, the sucrose application was taken up by rootstock seedlings, leading to higher starch accumulations in the grafted plants, leading to an increase in survival rates in polyploid watermelons. Carbohydrates are just as important hormones in the process of compatibility and callus formation, so are carbohydrates^49–53^. And it has been proven by the experiment that increasing the percentage of carbohydrates led to an increase in the rate of grafting survival^49^.

Overall, compatibility is a phenomenon that occurs through a combination of factors, including higher IAA, starch contents, and POD, CAT and SOD activities. All of these factors are more or less interrelated; this makes it easier for the plant to survive.

### Expression analysis of the IAA, ZR, antioxidants, biochemical substances, and transporter genes during the grafting process

During the grafting process, physiological changes lead to graft compatibility. It is important to identify the key genes and mechanisms in grafted watermelon to understand graft compatibility. IAA, ZR, and antioxidants have higher activity during the grafting process. From the results we can assume that *Cla97C05G092790* might play an important role in graft compatibility in tetraploid watermelon. The expressions of genes linked to POD, SOD, CAT, and starch increased at 3 DAG. These findings are similar to Ren et al.^19^ in cucumber and Liu et al.^36^ in citrus. The increased expression of Cla97C05G092790 leads to an increase in IAA signaling in tetraploid plants. Similarly, we observed higher expressions of genes linked to POD (*Cla97C07G135790, Cla97C01G019360),* SOD *(Cla97C04G071940, Cla97C10G187890),* CAT *(Cla97C08G152180),* and starch *Cla97C01G017330* at 3 DAG that leads to a higher compatibility. While expression of a gene linked to H_2_O_2_ (*Cla97C10G195960)* at 3 DAG decreased significantly. This study assumes that the survival rate of tetraploids is high due to high accumulation of hormones, antioxidants and starch and lower H_2_O_2_.

## Materials and methods

### Plant materials

Seedless watermelons are triploids (3n = 33) produced by crossing a tetraploid seed parent with a diploid (2n = 22) pollen parent. Tetraploid induction was done by applying colchicine to the growing apex of diploid seedlings. Polyploid seeds for one variety (*mimei*), which is homozygous and genetically stable and passed the achievement appraisal of the Chinese Department of Agriculture in 1990 and won the second prize of science technology progress of the Department of Agriculture in 1991^84^. Tetraploid, triploid, and diploid watermelons were grown for 60 days and used as mother plants. Branches were taken from these mother plants and grafted on squash interspecific (*C. maxima* × *C. moschata*) hybrid *(Xijiaqiangsheng*) widely used in China as a rootstock. Scions and rootstocks were obtained from the polyploid watermelon group—Zhengzhou Fruit research institute (CAAS) China. Our studies were complied with the relevant institutional, national, and international guidelines and legislation. The plant experiments were conducted according to local and national regulations and following Henan Joint International Research Laboratory of Fruits and Cucurbits Biological Science in South Asia and Zhengzhou Fruit research institute (CAAS) (Zhengzhou, China) regulations. All experiments were conducted in Zhengzhou Fruit Research Institute, Chinese Academy of Agricultural Sciences, Henan Joint International Research Laboratory of Fruits and Cucurbits Biological Science in South Asia Zhengzhou, China. We have permission to collect the Watermelon (*Citrullus lanatus* L.) plant, and the source of plants from the polyploid watermelon group—Zhengzhou Fruit research institute (CAAS) China.

**Figure 10:**
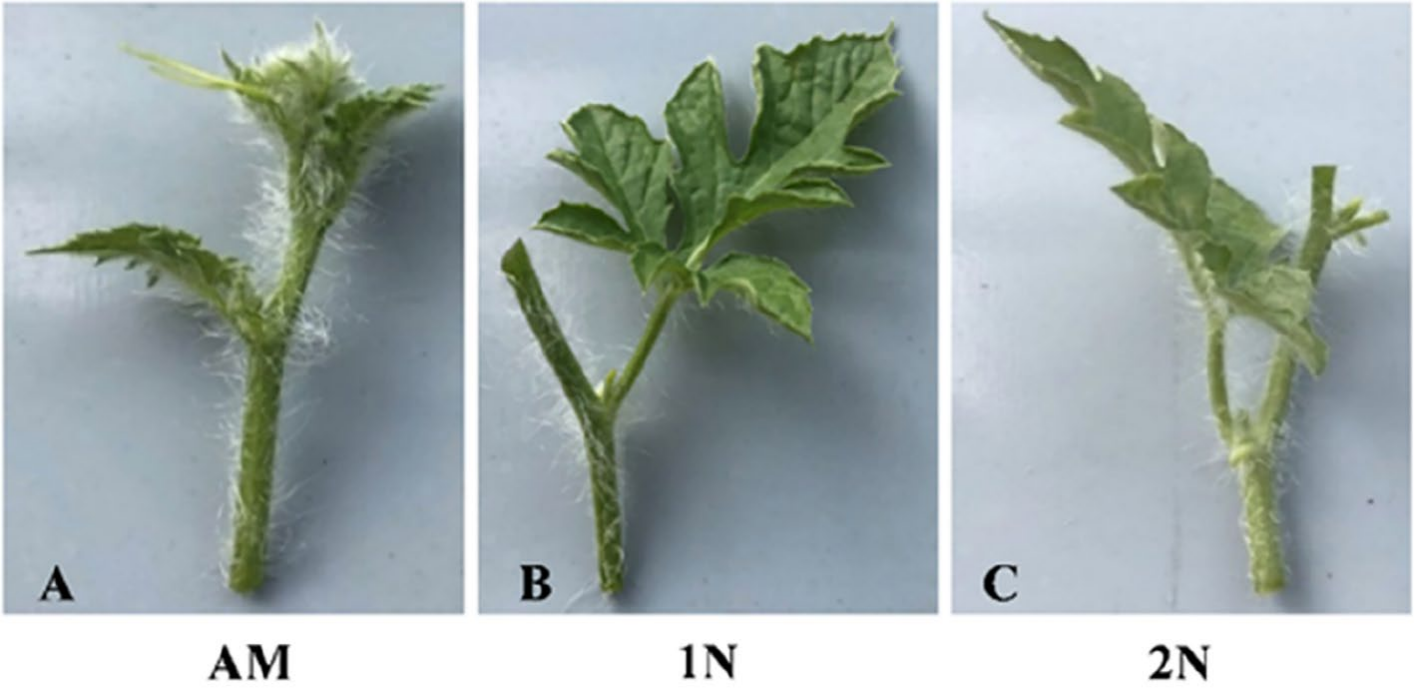
Three kinds of branches cutting were taken from mother plants to use as scions in diploid, triploid, and tetraploid watermelons, (**A**) Apical meristem (AM), (**B**) Branch having one node, and one leave (1N), and (**C**) Branch having two nodes (2N).

Tetraploid, triploid, and diploid watermelons and rootstock seeds were sown in seedling cell trays with 32 cells at the intelligent greenhouse of Zhengzhou, Henan province, China, during two consecutive seasons, August 2021 and March 2022. After 50–60 days after transplanting, good and healthy mother plants free of pests and diseases, especially virus-free, were selected for obtaining different types of scions. From the healthy mother plants, suitable branches were chosen for scions. Three types of branch cuttings (Fig. 10) were taken from mother plants, (a) apical meristem (AM), (b) the branch having one node and one leaf (1N), and (c) the branch having two nodes and one leaf (2N) as mentioned by El-Eslamboly^85^ and Khereba et al.^86^. The grafting process was performed after 15 and 20 days after rootstock seeds sowing. The splice grafting method was used^31,49^ (Fig. S1). Rootstock seedlings were subjected to adaptation before and after grafting to increase the survival rate, as mentioned by Oda^87,88^.

The samples [grafting combination (stems where scion and rootstock interlocked)] from the branch with one node (1N) were collected at three different stages 0, 3, and 15 days after grafting (DAG) for the determination of hormones, AOX, and RNA extraction. Each biological replicate sample from 30 plants was collected at three different stages, 0, 3, and 15 days after grafting (DAG). We collected 30 plants and considered them as one replicate. Samples were taken at 0 DAG as a control to study the grafting process development. Plants were cultivated in a growth chamber with 25–30 °C temperature and 60–85% humidity. The sensor in each experimental plot’s center was used to record temperature and humidity data (THtool-V151_En; Campbell Scientific Ltd., China).

### Sucrose application

According to the protocol established by sucrose or water applications to rootstocks seedlings, they were applied as a soil drench^49^. Sucrose solution was applied in the morning 2 and 4 days before grafting. Three replicates of 15 plants per treatment in the current experiment were maintained during two consecutive seasons. A randomized complete block design was used here with three replications and three biological replications.

### Data collection

#### Survival rates

Survival rates were determined after 15 days after grafting using the following formula^43^:

$$ {\text{Survival}}\;{\text{rate}} = \frac{{{\text{Number}}\;{\text{of}}\;{\text{survived}}\;{\text{grafted}}\;{\text{plants}}}}{{{\text{Total}}\;{\text{numbers}}\;{\text{of}}\;{\text{grafted}}\;{\text{plants}}}} \times 100 $$

#### Quantification of hormonal contents via ELISA

Samples were taken as three biological replicates from the grafting combination (stems where scion and rootstock interlocked) at 0, 3, and 15 days after grafting (DAG). The contents of IAA and Zeatin Riboside (ZR) were measured by the Enzyme-Linked Immunosorbent Assay (ELISA) method (College of Agriculture and Biotechnology, China Agricultural University). The determination of hormonal contents was performed as outlined by Mo et al.^25^.

#### Assay for antioxidants enzymes activity and H_2_O_2_ contents

SOD assay kit/YX-C-A500 was used for the measurement of superoxide dismutase activity at 560 nm wavelength. CAT assay kit/BC0200 for measuring catalase assay at 240 nm wavelength, POD assay kit/YX-C-A502 for measuring peroxidase activity at 570 nm wavelength, and H_2_O_2_ assay kit/YX-C-A400 for measuring Hydrogen peroxidase content at 415 nm wavelength (Sino best biological technology co, Ltd, Beijing, China) according to the manufacturer’s instructions. Three biological replications from grafting combination (stems where scion and rootstock interlocked) at 0, 3, and 15 DAG from three different plants for each replicate were collected for analysis. The activities of the antioxidant enzymes were expressed as a unit U g^−1^ FW sample.

#### Quantification of biochemical substances

Biochemicals were quantified by using the respective kits i.e., starch assay kit/YX-C-C400 for starch content estimation at 620 nm wavelength, lignin assay kit/YX-C-B636 for lignin content using 280 nm wavelength, and phenol assay kit/YX-C-A507 for total phenols content using 760 nm wavelength (Sino best biological technology co, Ltd, Beijing, China) according to the manufacturer’s instructions. Three biological replications from grafting combination (stems where scion and rootstock interlocked) at 0, 3, and 15 DAG from three different plants for each replicate were collected for analysis.

#### Identification of genes involved in the IAA, ZR, antioxidants, lignin, phenols, and starch metabolism and transport

The protein sequences of genes involved in IAA, ZR, antioxidants, lignin, phenols, and starch metabolism and transport in *Arabidopsis thaliana, Citrullus colocynthis, Cucumis melo, Cucumis sativus, Cucurbita maxima, Cucurbita moschata, Cucurbita pepo, Luffa aegyptiaca, and Momordica charantia* were downloaded from http://www.ncbi.nlm.nih.gov/Genbank/, https://phytozome.jgi.doe.gov/pz/portal.html, http://cucurbitgenomics.org/organism/2, and http://www.uniprot.org/ were used as queries for protein blast analysis against the Watermelon Reference Genome Database (http://cucurbitgenomics.org/organism/21) v2. MEGA X software was used to draw phylogenetic trees, the ClustalW tool was used to align protein sequences, and the neighbor-joining method with 1000 bootstrap replicates was applied to construct phylogenetic trees^55,89^.

#### Characteristics and structural analysis of genes associated with compatibility mechanisms in watermelon

Names of the 25 genes along with their accession numbers, genomic lengths, coding sequence lengths, protein sizes, and isoelectric points (pIs) and Mw (Da), were retrieved from two online tools, (1) http://cucurbitgenomics.org/organism/1 and (2) ExPASy http://web.expasy.org/computepi/ databases. An online tool namely “Gene Structure Display Server (GSDS)”, a web-based bioinformatics tool was used for the display of genes structures^90^.

#### Extraction of RNA and first-strand cDNA synthesis

According to the manufacturer’s instructions, total RNA was extracted from the grafting combination (stems where scion and rootstock interlocked) using RNA Kit (Tiangene, China). Samples from grafting combinations were taken at three stages (0, 3, and 15 days after grafting). 1% agarose gel was used to check RNA degradation and contamination. RNA quality and integrity were checked via NanoDrop ND-1000 spectrophotometer (Thermo Scientific, Wilmington, DE, USA) and an Agilent 2100 Bioanalyzer (Agilent Technologies, CA, USA). Extracted RNA was used for the cDNA synthesis for RT-qPCR, with M-MLV Reverse Transcriptase (Promega, USA).

#### RT-qPCR expression analysis of genes involved in compatibility mechanisms

RT-qPCR of genes linked to IAA, ZR, antioxidants, lignin, phenols, starch metabolism, and the transport was performed in grafted watermelon plants from the grafting combination (stems where scion and rootstock interlocked) at 0, 3, and 15 DAG (Fig. S4). Primer 3 was used to design primers for RTq-PCR analysis (Table S2). The entire data were analyzed using the 2^−ΔΔCt^ method^91^. Three independent biological replicates were used for gene expression analysis; Actin *“cla016178”* was used as a reference gene^55^.

#### Experimental design and statistical analysis

Data were statistically analyzed using XLSTAT PEARSON Edition (XLSTAT Version 2014.5.03) developed by Addinsoft. Excel for Microsoft 365 package version 16.0.14026.20246 used to draw all pictures. Analysis of variance (ANOVA) confirmed the significant variations among the split-plot design treatments with three replications. Duncan’s multiple range test (DMRT) compared the means sets at a 5% probability level.

## Conclusion

The current study concludes that vegetative propagation by branch grafting could be used to propagate triploid and tetraploid watermelons with high survival rates, making seed production easier and cheaper. An enigma of graft compatibility has been addressed here. Hormone, starch content and antioxidant activities were higher in tetraploids than in diploids due to genome duplication that produced high compatibility and high transplant survival rates. In addition, expression patterns of genes associated with biochemical and antioxidants also indicate their important role in the graft compatibility mechanisms. Using branch grafting can be an excellent alternative to encourage vegetative propagation in tetraploid watermelons. These results add important information to our existing knowledge of watermelon compatibility, ultimately aiding in vegetative propagation and breeding watermelon cultivars with desirable traits.

## Supplementary Information

Supplementary Information.

## Data Availability

All data available within the article and supplementary information file.
