# Supplementary material for: Comparative physiological and biochemical mechanisms in diploid, triploid, and tetraploid watermelon (Citrullus lanatus L.) grafted by branches
Source: Sci Rep. 2023 Mar 27;13:4993. doi: 10.1038/s41598-023-32225-z (PMC10043263; doi:10.1038/s41598-023-32225-z)
Supplement: Supplementary file 1 — Supplementary Information. [file 41598_2023_32225_MOESM1_ESM.pdf]

Supplementary material

Original research manuscript submitted to Scientific reports journal

Title: Comparative Physiological and Biochemical Mechanisms in Diploid, Triploid, and Tetraploid Watermelon (*Citrullus lanatus* L.) Grafted by Branches

Mohamed Omar Kaseb[1,2,*], Muhammad Jawad Umer[1,3], Xuqiang Lu[1], Nan He[1], Muhammad Anees[1], Eman El-remaly[2,4], Ahmed Fathy Yousef[5], Ehab A.A. Salama[6], Hazem M. Kalaji[7,8]and Wenge Liu[1,*]

[1] Zhengzhou Fruit Research Institute, Chinese Academy of Agricultural Sciences, Henan Joint International Research Laboratory of Fruits and Cucurbits Biological Science in South Asia Zhengzhou 450009, China.

[2] Horticulture Research Institute, Agriculture Research Center, Giza, 12611, Egypt.

[3] State Key Laboratory of Cotton Biology/Institute of Cotton Research, Chinese Academy of Agricultural Sciences (ICR, CAAS), Anyang 455000, China.

[4] Cross pollenated plants department, Horticulture Research Institute, Agriculture Research Center, Giza 12119, Egypt.

[5] Department of Horticulture, College of Agriculture, University of Al-Azhar (branch Assiut), Assiut 71524, Egypt.

[6] Agricultural Botany Department, Faculty of Agriculture Saba Basha, Alexandria University, 21531 Alexandria, Egypt.

[7] Department of Plant Physiology, Institute of Biology, Warsaw University of Life Sciences SGGW, Warsaw, Poland.

[8] Institute of Technology and Life Sciences, National Research Institute, Falenty, Al. Hrabska 3, 05-090 Raszyn, Poland.

* Corresponding author e-mail: liuwenge@caas.cn; mohamedkaseb@yahoo.com

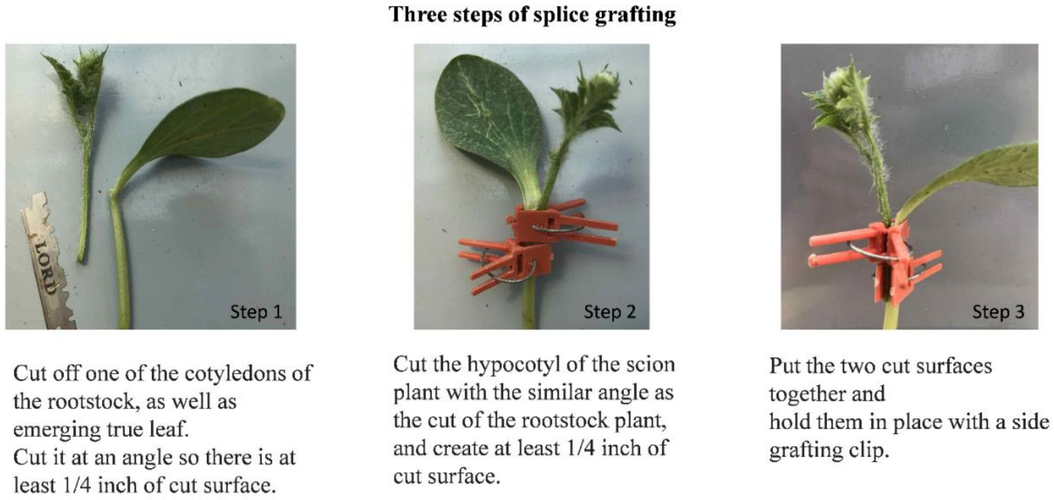

(Figure S1) Splice grafting method steps in polyploid watermelon plants

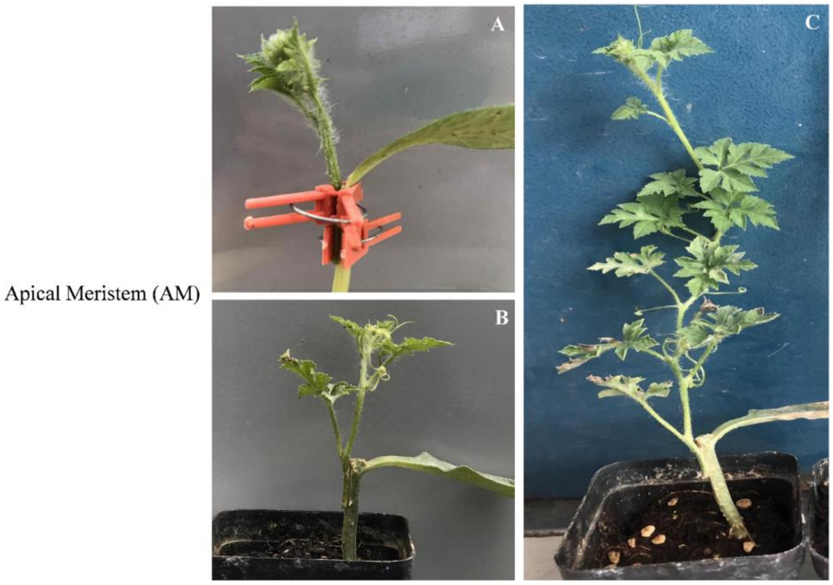

(Figure S2). Effect of Scion/rootstocks combinations on the survival of polyploid watermelon grafting using (AM) as scion. (A): at 0 DAG, (B) 15 DAG, and (C): 30 DAG, DAG: Days after Grafting.

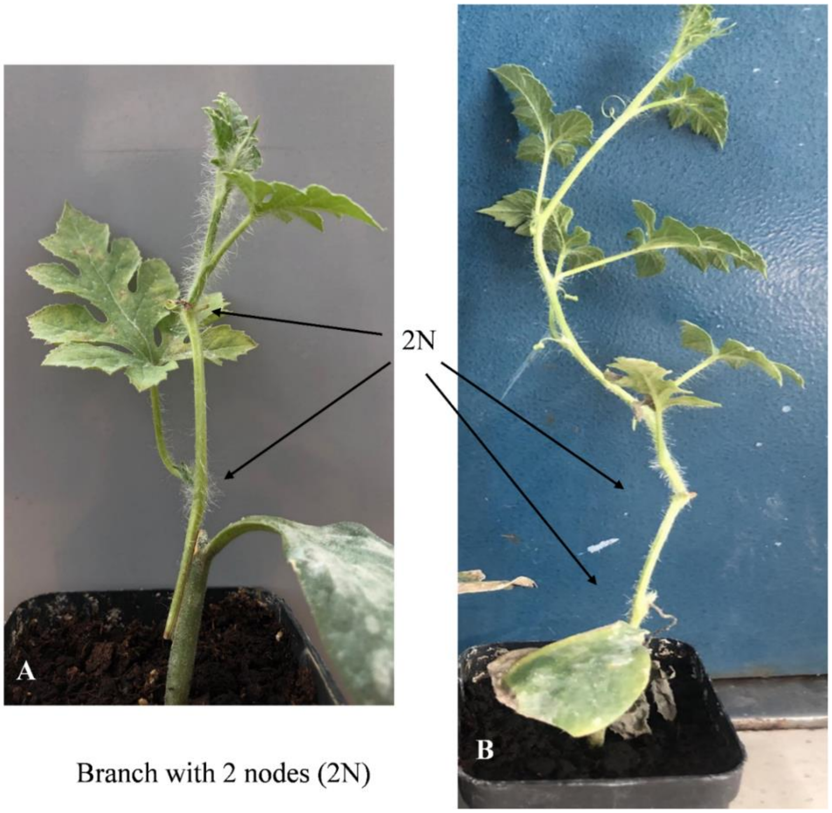

(Figure S3) Effect of Scion/rootstocks combinations on the survival of polyploid watermelon grafting using (2N) as scion. (A): at 15 DAG, and (B) 30 DAG.

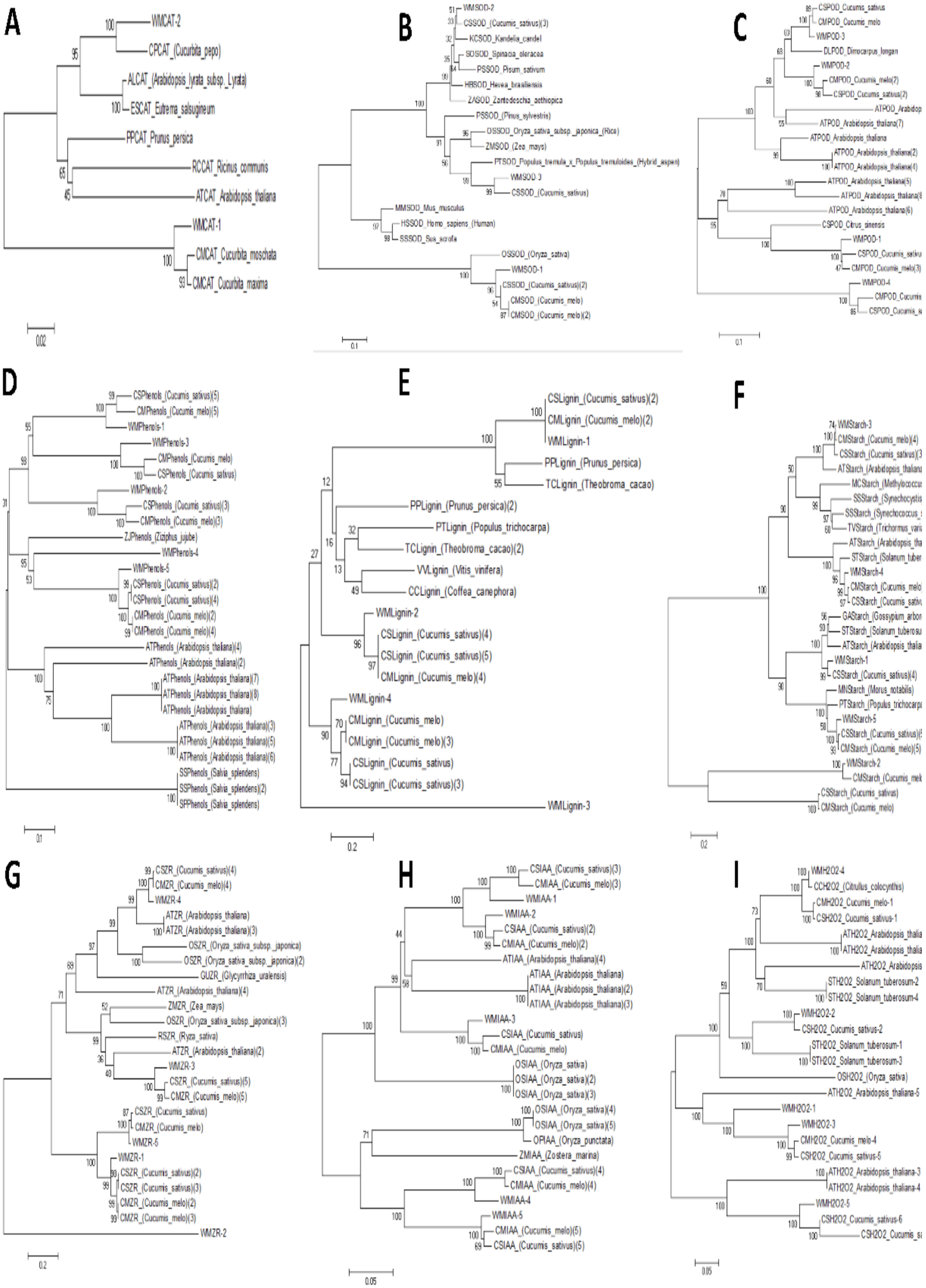

A
WMCAT-2
CPCAT_(Cucurbita_pepo)
ALCAT_(Arabidopsis_lyrata_subsp._Lyrata)
ESCAT_Eutrema_salsugineum
PPCAT_Prunus_persica
RCCAT_Ricinus_communis
ATCAT_Arabidopsis_thaliana
WMCAT-1
CMCAT_Cucurbita_moschata
CMCAT_Cucurbita_maxima
0.02
B
WMSOD-2
CSSOD_(Cucumis_sativus)(3)
KCSOD_Kandelia_candel
SOSOD_Spinacia_oleracea
PSSOD_Pisum_sativum
HBSOD_Hevea_brasiliensis
ZASOD_Zantedeschia_aethiopica
PSSOD_(Pinus_sylvestris)
OSSOD_Oryza_sativa_subsp._japonica_(Rice)
ZMSOD_(Zea_mays)
PTSOD_Populus_tremula_x_Populus_tremuloides_(Hybrid_aspen)
WMSOD-3
CSSOD_(Cucumis_sativus)
MMSOD_Mus_musculus
HSSOD_Homo_sapiens_(Human)
SSSOD_Sus_scrofa
OSSOD_(Oryza_sativa)
WMSOD-1
CSSOD_(Cucumis_sativus)(2)
CMSOD_(Cucumis_melo)
CMSOD_(Cucumis_melo)(2)
0.1
C
CSPOD_Cucumis_sativus
CMPOD_Cucumis_melo
WMPOD-3
DLPOD_Dimocarpus_longan
WMPOD-2
CMPOD_Cucumis_melo(2)
CSPOD_Cucumis_sativus(2)
ATPOD_Arabidop
ATPOD_Arabidopsis_thaliana(7)
ATPOD_Arabidopsis_thaliana
ATPOD_Arabidopsis_thaliana(2)
ATPOD_Arabidopsis_thaliana(4)
ATPOD_Arabidopsis_thaliana(5)
ATPOD_Arabidopsis_thaliana(6)
CSPOD_Citrus_sinensis
WMPOD-1
CSPOD_Cucumis_sativu
CMPOD_Cucumis_melo(3)
WMPOD-4
CMPOD_Cucumis
CSPOD_Cucumis_s
0.1
D
CSPhenols_(Cucumis_sativus)(5)
CMPhenols_(Cucumis_melo)(5)
WMPhenols-1
WMPhenols-3
CMPhenols_(Cucumis_melo)
CSPhenols_(Cucumis_sativus)
WMPhenols-2
CSPhenols_(Cucumis_sativus)(3)
CMPhenols_(Cucumis_melo)(3)
ZJPhenols_(Ziziphus_jujube)
WMPhenols-4
WMPhenols-5
CSPhenols_(Cucumis_sativus)(2)
CSPhenols_(Cucumis_sativus)(4)
CMPhenols_(Cucumis_melo)(2)
CMPhenols_(Cucumis_melo)(4)
ATPhenols_(Arabidopsis_thaliana)(4)
ATPhenols_(Arabidopsis_thaliana)(2)
ATPhenols_(Arabidopsis_thaliana)(7)
ATPhenols_(Arabidopsis_thaliana)(8)
ATPhenols_(Arabidopsis_thaliana)
ATPhenols_(Arabidopsis_thaliana)(3)
ATPhenols_(Arabidopsis_thaliana)(5)
ATPhenols_(Arabidopsis_thaliana)(6)
SSPhenols_(Salvia_splendens)
SSPhenols_(Salvia_splendens)(2)
SPPhenols_(Salvia_splendens)
0.1
E
CSLignin_(Cucumis_sativus)(2)
CMLignin_(Cucumis_melo)(2)
WMLignin-1
PPLignin_(Prunus_persica)
TCLignin_(Theobroma_cacao)
PPLignin_(Prunus_persica)(2)
PTLignin_(Populus_trichocarpa)
TCLignin_(Theobroma_cacao)(2)
VVLignin_(Vitis_vinifera)
CCLignin_(Coffea_canephora)
WMLignin-2
CSLignin_(Cucumis_sativus)(4)
CSLignin_(Cucumis_sativus)(5)
CMLignin_(Cucumis_melo)(4)
WMLignin-4
CMLignin_(Cucumis_melo)
CMLignin_(Cucumis_melo)(3)
CSLignin_(Cucumis_sativus)
CSLignin_(Cucumis_sativus)(3)
WMLignin-3
0.2
F
WMStarch-3
CMStarch_(Cucumis_melo)(4)
CSStarch_(Cucumis_sativus)(3
ATStarch_(Arabidopsis_thaliana
MCStarch_(Methylococcus
SSStarch_(Synechocystis
SSStarch_(Synechococcus_
TVStarch_(Trichormus_vari
ATStarch_(Arabidopsis_tha
STStarch_(Solanum_tuber
WMStarch-4
CMStarch_(Cucumis_melo
CSStarch_(Cucumis_sativ
GAStarch_(Gossypium_arbor
STStarch_(Solanum_tuberosu
ATStarch_(Arabidopsis_thalia
WMStarch-1
CSStarch_(Cucumis_sativus)(4)
MNStarch_(Morus_notabilis)
PTStarch_(Populus_trichocarpa
WMStarch-5
CSStarch_(Cucumis_sativus)(
CMStarch_(Cucumis_melo)(5)
WMStarch-2
CMStarch_(Cucumis_mel
CSStarch_(Cucumis_sativus)
CMStarch_(Cucumis_melo)
0.2
G
CSZR_(Cucumis_sativus)(4)
CMZR_(Cucumis_melo)(4)
WMZR-4
ATZR_(Arabidopsis_thaliana)
ATZR_(Arabidopsis_thaliana)(3)
OSZR_(Oryza_sativa_subsp._japonica)
OSZR_(Oryza_sativa_subsp._japonica)(2)
GUZR_(Glycyrrhiza_uralensis)
ATZR_(Arabidopsis_thaliana)(4)
ZMZR_(Zea_mays)
OSZR_(Oryza_sativa_subsp._japonica)(3)
RSZR_(Ryza_sativa)
ATZR_(Arabidopsis_thaliana)(2)
WMZR-3
CSZR_(Cucumis_sativus)(5)
CMZR_(Cucumis_melo)(5)
CSZR_(Cucumis_sativus)
CMZR_(Cucumis_melo)
WMZR-5
WMZR-1
CSZR_(Cucumis_sativus)(2)
CSZR_(Cucumis_sativus)(3)
CMZR_(Cucumis_melo)(2)
CMZR_(Cucumis_melo)(3)
WMZR-2
0.2
H
CSIAA_(Cucumis_sativus)(3)
CMIAA_(Cucumis_melo)(3)
WMIAA-1
WMIAA-2
CSIAA_(Cucumis_sativus)(2)
CMIAA_(Cucumis_melo)(2)
ATIAA_(Arabidopsis_thaliana)(4)
ATIAA_(Arabidopsis_thaliana)
ATIAA_(Arabidopsis_thaliana)(2)
ATIAA_(Arabidopsis_thaliana)(3)
WMIAA-3
CSIAA_(Cucumis_sativus)
CMIAA_(Cucumis_melo)
OSIAA_(Oryza_sativa)
OSIAA_(Oryza_sativa)(2)
OSIAA_(Oryza_sativa)(3)
OSIAA_(Oryza_sativa)(4)
OSIAA_(Oryza_sativa)(5)
OPIAA_(Oryza_punctata)
ZMIAA_(Zostera_marina)
CSIAA_(Cucumis_sativus)(4)
CMIAA_(Cucumis_melo)(4)
WMIAA-4
WMIAA-5
CMIAA_(Cucumis_melo)(5)
CSIAA_(Cucumis_sativus)(5)
0.05
I
WMH2O2-4
CCH2O2_(Citrullus_colocynthis)
CMH2O2_Cucumis_melo-1
CSH2O2_Cucumis_sativus-1
ATH2O2_Arabidopsis_thalia
ATH2O2_Arabidopsis_thalia
ATH2O2_Arabidopsis
STH2O2_Solanum_tuberosum-2
STH2O2_Solanum_tuberosum-4
WMH2O2-2
CSH2O2_Cucumis_sativus-2
STH2O2_Solanum_tuberosum-1
STH2O2_Solanum_tuberosum-3
OSH2O2_(Oryza_sativa)
ATH2O2_Arabidopsis_thaliana-5
WMH2O2-1
WMH2O2-3
CMH2O2_Cucumis_melo-4
CSH2O2_Cucumis_sativus-5
ATH2O2_Arabidopsis_thaliana-3
ATH2O2_Arabidopsis_thaliana-4
WMH2O2-5
CSH2O2_Cucumis_sativus-6
CSH2O2_Cucumis_sa
0.05

Figure S4: Maximum likelihood phylogeny of watermelon genes encoding key enzymes and transporters involved graft compatibility with those from *Arabidopsis lyrata* subsp. *Lyrata*, *Arabidopsis thaliana, Citrullus colocynthis, Citrus sinensis, Coffea canephora, Cucumis melo, Cucumis sativus, Cucurbita maxima, Cucurbita moschata, Cucurbita pepo, Dimocarpus longan, Eutrema salsugineum, Glycyrrhiza uralensis, Gossypium arboretum, Hevea brasiliensis, Kandelia candel, Morus notabilis, Oryza punctate, Oryza sativa, Pinus Sylvestris, Pisum sativum, Populus tremula x Populus tremuloides* (Hybrid aspen), *Populus trichocarpa, Prunus persica, Ricinus communis, Salvia splendens, Solanum tuberosum, Theobroma cacao, Vitis vinifera, Zantedeschia aethiopica, Zea mays, Ziziphus jujube, and Zostera marina*. The phylogenetic tree was constructed from protein sequences using the neighbor-joining method.

Table S1: Genes linked to graft compatibility having high homology with other genes selected from different plant species.

		High homology
WMCAT	WMCAT-1	CMCAT_*Cucurbita moschata*<br>CMCAT_*Cucurbita_maxima*
	WMCAT-2	CPCAT_*Cucurbita pepo*.
WMPOD	WMPOD-1	CSPOD_*Cucumis sativus3*<br>CMPOD_*Cucumis melo3*.
	WMPOD-2	CMPOD_*Cucumis melo*2<br>CSPOD_*Cucumis sativus*2
	WMPOD-3	CMPOD_*Cucumis melo*<br>CSPOD_*Cucumis sativus*
	WMPOD-4	CMPOD_*Cucumis melo*4 and CSPOD_*Cucumis sativus*4
WMIAA	WMIAA-1	CSIAA_*Cucumis sativus3* and CMIAA_*Cucumis melo3*
	WMIAA-2	CSIAA_*Cucumis sativus*2 and CMIAA_*Cucumis melo*2
	WMIAA-3	CSIAA_*Cucumis sativus* and CMIAA_*Cucumis melo*
	WMIAA-4	CMIAA_*Cucumis melo*4 and CSIAA_*Cucumis sativus*4
	WMIAA-5	CMIAA_*Cucumis melo*5 and CSIAA_*Cucumis sativus*5
WMZR	WMZR-1	CSZR_*Cucumis sativus2*, CSZR_*Cucumis sativus*3 CMZR_*Cucumis melo*2, and CMZR_*Cucumis melo*3
	WMZR-2	-
	WMZR-3	CSZR_*Cucumis sativus*5 and CMZR_*Cucumis melo*5
	WMZR-4	CMZR_*Cucumis melo*4 and CSZR_*Cucumis sativus*4
	WMZR-5	CMZR_*Cucumis melo* and CSZR_*Cucumis sativus*
WMPhenols	WMPhenols-1	CSPhenols_*Cucumis sativus5* and CMPhenols_*Cucumis melo5*
	WMPhenols-2	CSPhenols_*Cucumis sativus*3 and CMPhenols_*Cucumis melo*3
	WMPhenols-3	CMPhenols_*Cucumis melo*, and CSPhenols_*Cucumis sativus*.
	WMPhenols-4	-
	WMPhenols-5	CMPhenols_*Cucumis melo*2, CMPhenols_*Cucumis melo*4<br>CSPhenols_*Cucumis sativus*2, CSPhenols_*Cucumis sativus*4.
WMLignin	WMLignin-1	CSLignin_*Cucumis sativus* and CMLignin_*Cucumis melo*
	WMLignin-2	CMLignin_*Cucumis melo*4, CSLignin_*Cucumis sativus*4, and CSLignin_*Cucumis sativus*5.
	WMLignin-3	-
	WMLignin-4	CMLignin_*Cucumis melo*1, CMLignin_*Cucumis melo*3, CSLignin_*Cucumis sativus*1, and CSLignin_*Cucumis sativus3*.
	WMStarch-1	CSStarch_*Cucumis sativus4*.

WMStarch	WMStarch-2	CMStarch_*Cucumis melo*2
	WMStarch-3	CMStarch_*Cucumis melo4*, and CSStarch_*Cucumis sativus3*.
	WMStarch-4	CMStarch_*Cucumis melo3*, and CSStarch_*Cucumis sativus2*.
	WMStarch-5	CMStarch_*Cucumis melo5*, and CSStarch_*Cucumis sativus5*
WMH$_2$O$_2$	WMH$_2$O$_2$-1	CSH$_2$O$_2$_*Cucumis sativus5* and CMH$_2$O$_2$_*Cucumis melo*4
	WMH$_2$O$_2$-2	CSH$_2$O$_2$_*Cucumis sativus2*.
	WMH$_2$O$_2$-3	CSH$_2$O$_2$_*Cucumis sativus5* and CMH$_2$O$_2$_*Cucumis melo*4
	WMH$_2$O$_2$-4	CCH$_2$O$_2$_*Citrullus colocynthis*.
	WMH$_2$O$_2$-5	CSH$_2$O$_2$_*Cucumis sativus6*, and CSH$_2$O$_2$_*Cucumis sativus7*.
WMSOD	WMSOD-1,	CSSOD_*Cucumis sativus2*, CMSOD_*Cucumis melo*, and CMSOD_*Cucumis melo*2.
	WMSOD-2	CSSOD_*Cucumis sativus3*.
	WMSOD-3	CSSOD_*Cucumis sativus*

Table *S2*: Primers used for qRT-PCR in this study.

ID	primer
Actin-F	GTACGACAACGGGCCTTAAA
Actin-R	ATGGGCTTGACAGGTTGTTC
WMCAT-1-F	CTAATGTGTTGAGCGGCAAA
WMCAT-1-R	GTCTTACGAACCGCTCTTGC
WMCAT-2-F	CGGCATTACTAAAGCGGAAG
WMCAT-2-R	TAGTCCAGAAGGGGGTGTTG
WMPOD-1-F	TTGGATGCCTACACGATCAA
WMPOD-1-R	GTATTGGCCAGCTTCACCAT
WMPOD-2-F	CATTGGGAAGGAAGGACTCA
WMPOD-2-R	CAATGTCAAGCCCTTGGTTT
WMPOD-3-F	GCTCTTTCAGGAAGCCACAC
WMPOD-3-R	GGTAGGGCTCACAAAGTCCA
WMPOD-4-F	TGGCTGTGATGCTTCTATGC
WMPOD-4-R	CTGCACAAGAGACGATTCCA
WMSOD-1-F	TTTCTAAGGTCCACCGCAAC
WMSOD-1-R	GGGAATTGGGTTTGAAGGAT
WMSOD-2-F	CAAGAAGACGATGGAGCACA
WMSOD-2-R	TCCATCAGCATTGGCAATTA
WMSOD-3-F	CCATCCAGTTCGTCCAAGAT
WMSOD-3-R	CCTCCATGGTCCTTCTTCAA
WMH2O2-1-F	GGGTTACTTCGGACCAGACA

WMH2O2-1-R	GAAACCCGTCAGCCTATCAA
WMH2O2-2-F	ATGACCAAAAACGTCGGAAG
WMH2O2-2-R	ACTCGCTCGACTCGTTCATT
WMH2O2-3-F	AACTTCTGCTTCGACCTCCA
WMH2O2-3-R	AACTCGTGCGAGAAATGCTT
WMH2O2-4-F	AGCCAACAAACGAAACCAAC
WMH2O2-4-R	TCTGTGGCGATATTGGATGA
WMH2O2-5-F	CAGTCCAGGTTCCATTCGTT
WMH2O2-5-R	TTCTCAACGCGAACTCCTTT
WMIAA-1-F	AATGTCGAGTGGCCGATAAC
WMIAA-1-R	GCACCGTGGTGCCTTATACT
WMIAA-2-F	TTCCCACGCTGGAATTTTAC
WMIAA-2-R	GGGAGGAATTCGAAGTAGCC
WMIAA-3-F	ACCTCTATGTGCCTGGGTTG
WMIAA-3-R	GGCAAAGAATGGCCTCATTA
WMIAA-4-F	GGTGAATTTGAAGCCGATGT
WMIAA-4-R	CAAGCTCCTTTTGCAACTCC
WMIAA-5-F	AACAATTCCAGGCCACTACG
WMIAA-5-R	CCCTGTCGGTATACGCTGTT
WMZR-1-F	GACCGCCTATTGCCTCATTA
WMZR-1-R	TTCCCACACACATTGCTGTT
WMZR-2-F	CGCATTATCCCAGCTCTCAT
WMZR-2-R	TAGCGAGACTGAGCGAGACA
WMZR-3-F	GTTAGCGAGGGGAGCTTTCT
WMZR-3-R	AGCACTCTTTTTGGCGTTGT
WMZR-4-F	GTGTCGTGGACATGTTGGAG
WMZR-4-R	GCTAGAGCCAAATGCTGTCC
WMZR-5-F	GTTACGCGGGGTATATGGTG
WMZR-5-R	GCTGCTTTCAAACTCGGTTC
WMLignin-1-F	CCTTTATGGGGGAATCCTGT
WMLignin-1-R	GTGAACAAAAACGGGTTGCT
WMLignin-2-F	ACTCGTTGCCTTTGTCTCGT
WMLignin-2-R	GAAACTGCAACTCGTGCAAA
WMLignin-3-F	TATGGAAAAGGATGGGTGGA
WMLignin-3-R	CTTTAACGTGGCTCGTCCTC
WMLignin-4-F	GAGTCGGAGGTTTGGTGTGT
WMLignin-4-R	CCGACCAAGAGATGAGAAGG

WMPhenols-1-F	GGCTCATAAATCCCCCATTT
WMPhenols-1-R	TGCAGGTTGTTGTCCAGTGT
WMPhenols-2-F	CTCACTATGTCGCCGGATTT
WMPhenols-2-R	ACCGTGAAGGTGGAGAGATG
WMPhenols-3-F	AAGGTGCCTGCTTCACCTAA
WMPhenols-3-R	TATTTCCCCAACCAAACCAA
WMPhenols-4-F	CGATTGAGCAACAAAAAGCA
WMPhenols-4-R	CTGTAGGAGGGCTCACGAAC
WMPhenols-5-F	CCCGATACTCATCGTTCGTT
WMPhenols-5-R	GGCGTAGAAACCCAGGTACA
WMStarch-1-F	TGATTGGCACACAGCTCTTC
WMStarch-1-R	AATTGATCGGGCAGATTCAG
WMStarch-2-F	GCAAAATTTTCCCGAGATGA
WMStarch-2-R	TTTTATTCTCGCACGCCTCT
WMStarch-3-F	GCTCTGGCTTTGGTTCTTTG
WMStarch-3-R	GTCGCATTTTACACGAAGCA
WMStarch-4-F	TTTGATCATGGGAGCATTCA
WMStarch-4-R	GCCTCTTTGGGAACATGAAA
WMStarch-5-F	AAAGGACGGACAACCAAGTG
WMStarch-5-R	GCCAGCTACGTCTCCAAGTC
